# Preventative semaglutide and tirzepatide treatment does not alter disease progression in the 5xFAD mouse model of Alzheimer's disease

**DOI:** 10.1016/j.xcrm.2026.102906

**Published:** 2026-07-07

**Authors:** Anika Vear, Sofie Amalie Olsen, Emilie Cathrine Holst Lange, Marie Amalie Müller, Charlotte Svendsen, Christoffer Clemmensen

**Affiliations:** 1 Novo Nordisk Foundation Centre for Basic Metabolic Research, University of Copenhagen, 2200 Copenhagen, Denmark

**Keywords:** Alzheimer's disease, 5xFAD, LPS, GLP-1, GIP, incretin hormones, semaglutide, tirzepatide

## Abstract

There is growing evidence that long-acting mimetics of the gut-derived incretin hormones GLP-1 and GIP act as disease-modifying therapies for Alzheimer's disease (AD). Here, we temporally characterize the efficacy of the approved incretin receptor agonists semaglutide, a GLP-1R agonist, and tirzepatide, a GLP-1R/GIPR co-agonist, in preventing AD progression. In 5xFAD mice treated for 2 or 4 months, both incretin therapies lower body weight and improve glucose tolerance, yet neither compound produces measurable effects on memory or learning tasks, amyloid-β plaque deposition, or glial cell activation. In a non-amyloidogenic model, 3 days of incretin pre-treatment does not alter microglial activation or the expression of inflammatory markers following lipopolysaccharide (LPS) administration in mice. Our findings indicate that chronic semaglutide or tirzepatide treatment, even when initiated before overt pathology and delivered for a prolonged period, does not slow neuropathological progression in 5xFAD or LPS-treated mice.

## Highlights

• Semaglutide and tirzepatide improve metabolic dysfunction in 5xFAD mice
• Incretin therapy does not alter the progression of AD neuropathology in 5xFAD mice
• Neuroinflammation is not prevented by incretin therapy in LPS-treated mice

## Graphical abstract

**Figure:**
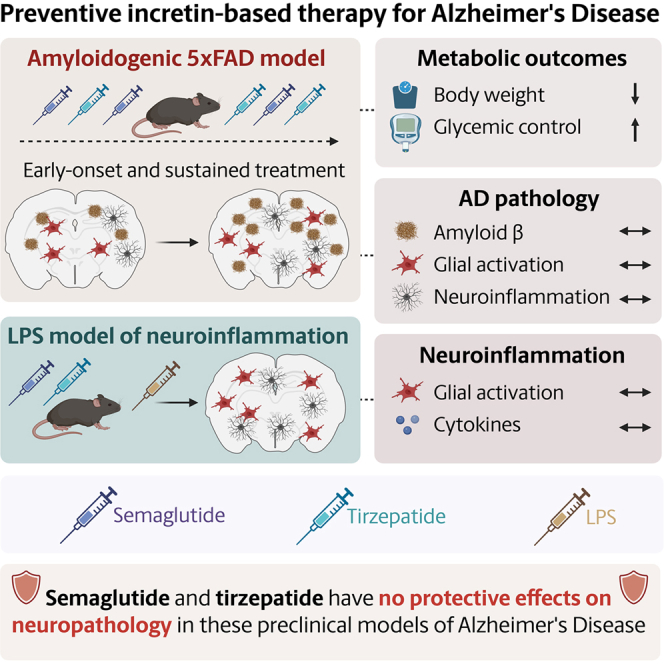

## Teaser

Vear et al. report that chronic semaglutide or tirzepatide treatment does not slow the progression of Alzheimer's disease neuropathology in two distinct mouse models, the 5xFAD mouse or lipopolysaccharide-treated mice. This guides the design of future preclinical investigations testing next-generation incretin mimetics for brain diseases.

## Introduction

Alzheimer’s disease (AD) is a debilitating neurodegenerative disease that affects more than 50 million people worldwide.^1^ It is characterized by a gradual decline in memory and cognitive functions, which severely diminishes patient quality of life. As the disease advances, neuropsychiatric symptoms such as depression and anxiety appear as well as a loss of language and motor functions, with complications associated with the disease such as pneumonia resulting in death.^1^

In recent years, the first disease-modifying therapies for AD have emerged in the form of passive immunotherapies targeting one of the classical hallmarks of AD—the accumulation of insoluble toxic amyloid-beta (Aβ) peptide aggregates.^2^ Although these therapies can slow the progression of Aβ pathology, their benefits are limited, and they are associated with potential adverse effects in some AD subpopulations.^2^ Moreover, they only address one pathological feature of the disease, thereby limiting their potential clinical efficacy. AD is characterized by a range of interconnected hallmarks beyond the deposition of Aβ plaques, including tau-containing neurofibrillary tangles, neuroinflammation, central metabolic dysregulation, mitochondrial dysfunction, impaired autophagy, and neuronal senescence.^3^ Therapeutic strategies that target multiple of these hallmarks hold significant promise for improving the efficacy of future AD therapies.

Glucagon-like peptide-1 receptor (GLP-1R) agonists have had a groundbreaking impact on the management of cardiometabolic diseases with their broad protective actions across multiple organ systems attributed to pleiotropic effects on diverse metabolic and inflammatory pathways.^4^^,^^5^ Importantly, these protective actions also extend to the central nervous system (CNS) where they are currently being investigated for the treatment of neuropsychiatric conditions such as alcohol-use disorder and major depressive disorder and neurodegenerative diseases such as Parkinson's disease and AD.^4^ In support of these central protective effects, a recent epidemiological study that pooled three large double-blind, randomized, controlled trials using the GLP-1R agonists liraglutide (LEADER), subcutaneously administered semaglutide (SUSTAIN-6), and oral semaglutide (PIONEER-6) revealed a lower incidence of dementia in people with type II diabetes randomized to a GLP-1R agonist treatment for 5 years compared with placebo.^6^ Semaglutide is also the first GLP-1R agonist to enter phase III clinical evaluation for mild cognitive impairment and early AD (EVOKE trial NCT04777396, EVOKE+ trial NCT04777409). However, recently announced topline results indicate that semaglutide did not demonstrate superiority over placebo in slowing clinical progression, measured as change in Clinical Dementia Rating-Sum of Boxes over 156 weeks, although some AD-related biomarkers in the cerebrospinal fluid were modulated.^7^

In preclinical rodent models, GLP-1R agonists have been found to increase glucose uptake, decrease neuroinflammation, promote synaptic plasticity, decrease neuronal excitotoxicity and apoptosis, and decrease oxidative stress across disease models of neurodegeneration, extensively reviewed previously.^4^ Early-generation GLP-1R agonists, such as exenatide and liraglutide, have also shown therapeutic potential in rodent models of AD, including the double transgenic APP/PS1 mouse model,^8^^,^^9^ triple transgenic APP/PS1/Tau mouse model,^10^ 5xFAD mouse model,^11^^,^^12^ aggregated Aβ_1–42_-injected mouse and rat models,^13^^,^^14^^,^^15^ streptozotocin (STZ)-induced mouse model,^16^ and senescence-accelerated mouse model.^17^

More recently, treatment with the GLP-1R agonist, semaglutide, improved peripheral glycemic control, Aβ plaque load, neuroinflammation, and learning and spatial memory in 7- and 12-month-old APP/PS1/Tau triple transgenic mice^18^^,^^19^ and 6-month-old APP/PS1 double transgenic mice.^20^ However, Forny Germano et al.^21^ reported that 6–7 weeks of treatment with semaglutide or the GLP-1R/glucose-dependent insulinotropic peptide receptor (GIPR) dual agonist, tirzepatide, does not reverse AD pathology or improve behavior in 6- or 12-month-old 5xFAD or APP/PS1 mouse models. These findings highlight that incretin-based therapies may have limited impact once pathology is well established, leaving open the question of whether earlier intervention could exert protective effects.

Therefore, in this study, we evaluated the potential preventative actions of molar-matched doses of semaglutide and tirzepatide in the 5xFAD mouse model. We examined metabolic and behavioral outcomes, whole-brain Aβ plaque deposition, and region-specific inflammatory and synaptic markers to assess whether early, sustained incretin therapy can modify disease-relevant processes. Additionally, the protective actions of these drugs on inflammatory processes were tested in the lipopolysaccharide (LPS)-induced mouse model of neuroinflammation, as an alternate non-transgenic model with a different disease pathogenesis and trajectory.

## Results

### Metabolic actions of semaglutide and tirzepatide treatment in 5xFAD mice

**Figure 1:**
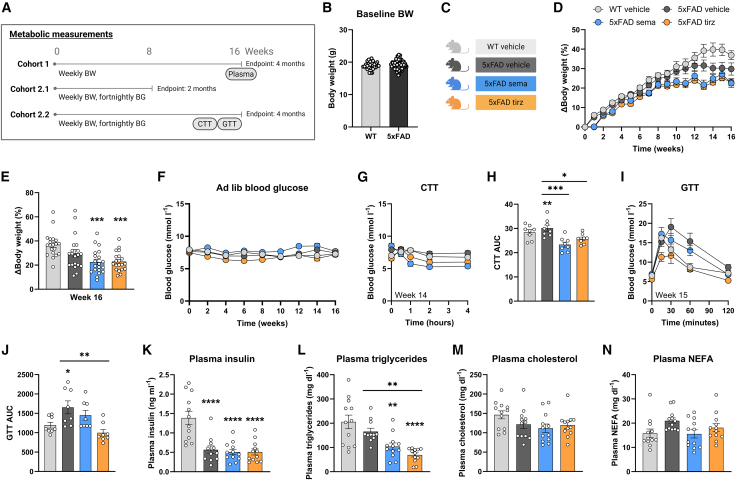
Metabolic actions of semaglutide and tirzepatide treatment in 5xFAD mice (A) Eight-week-old female WT or 5xFAD mice, (B) which had similar body weights (BW; g) at baseline, were treated on alternate days with either (C) vehicle, semaglutide (10 nmol kg^−1^) or tirzepatide (10 nmol kg^−1^) for 2 or 4 months. (D) Weekly percent change in body weight from baseline in mice across all cohorts (*n* = 20–28). (E) Percent change in body weight from baseline at endpoint (week 16) (*n* = 20). (F) Fortnightly *ad libitum* blood glucose (mmol L^−1^) measurements (n = 8–16). (G) Blood glucose (mmol L^−1^) measurements at 0, 0.5, 1, 2, and 4 h post-injection and (H) area under the curve (AUC) in a compound tolerance test (CTT) conducted at week 14 (*n* = 8). (I) Blood glucose (mmol L^−1^) measurements at 0, 15, 30, 60, and 120 min after an intraperitoneal (i.p.) glucose injection and (J) AUC in a glucose tolerance test (GTT) conducted at week 15 (*n* = 8). Plasma analysis of (K) insulin (ng mL^−1^), (L) triglycerides (mg dL^−1^), (M) cholesterol (mg dL^−1^), and (N) NEFA (mg dL^−1^) at endpoint in mice from cohort 1 (*n* = 12). Data are presented as mean ± SEM. ∗*p* < 0.05, ∗∗*p* < 0.01, ∗∗∗*p* < 0.001, ∗∗∗∗*p* < 0.0001 vs. WT vehicle (unless specified) via one-way ANOVA with Tukey’s post-hoc test. See also Figure S2A.

Given the potent cardiometabolic actions of GLP-1R agonists on glucose and body weight regulation, various metabolic parameters were assessed in semaglutide- and tirzepatide-treated 5xFAD mice (Figures 1A and S1). At baseline, body weight was comparable across the wild-type (WT) and 5xFAD genotypes (Figure 1B). Within the first two days of treatment, tirzepatide attenuated body weight gain, as expected, whereas semaglutide-treated 5xFAD mice had similar acute body weight changes to both WT vehicle and 5xFAD vehicle groups (Figure S2A). Body weight was subsequently recorded weekly across all cohorts for the entire treatment duration (Figure 1C). After 16 weeks of treatment, the gain in body weight from baseline of 5xFAD mice treated with semaglutide (22.6% ± 2.3%) or tirzepatide (22.9% ± 1.8%) was significantly lower than the vehicle-treated 5xFAD mice (29.8% ± 3.1%) (Figures 1D and 1E).

Fortnightly *ad libitum* blood glucose measurements were comparable across all groups in cohort 2 (Figure 1F). In a compound tolerance test (conducted at week 14 of treatment), semaglutide and tirzepatide induced significant decreases in acute blood glucose levels post-injection compared to vehicle-treated controls (Figures 1G and 1H). Notably, there was also an increase in acute blood glucose following injection with the vehicle in 5xFAD mice compared to WT mice (Figures 1G and 1H). In a glucose tolerance test (conducted at week 15 of treatment), tirzepatide fully normalized the dysfunction in glucose tolerance observed in 5xFAD vehicle mice to levels comparable to that of the WT vehicle group (Figures 1I and 1J). This response was only partially recovered in semaglutide-treated mice (Figures 1I and 1J).

Finally, circulating levels of different metabolic markers were examined in the plasma of cohort 1 after 16 weeks of treatment. All 5xFAD mice regardless of treatment had similar 3-fold decreases in plasma insulin levels compared to WT vehicle-treated mice (Figure 1K). GLP-1R agonist treatment also significantly reduced plasma triglycerides 2- to 3-fold compared to the WT vehicle group and 2-fold compared to the 5xFAD vehicle group for tirzepatide only (Figure 1L). No significant genotype or drug effects were observed in plasma cholesterol (Figure 1M) or non-esterified fatty acids (NEFAs) (Figure 1N), as has been previously reported.^22^^,^^23^

### Behavioral effects of semaglutide and tirzepatide treatment in 5xFAD mice

**Figure 2:**
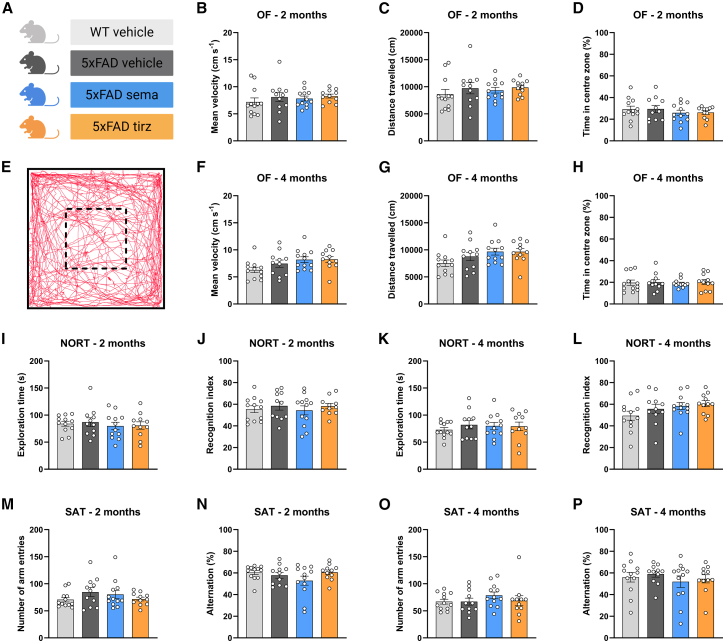
Behavioral effects of semaglutide and tirzepatide treatment in 5xFAD mice (A–P) (A) Eight-week-old female WT or 5xFAD mice in cohort 1 were treated on alternate days with either vehicle, semaglutide (10 nmol kg^−1^), or tirzepatide (10 nmol kg^−1^) and underwent behavioral testing after 2 and 4 months of treatment. (B–H) In the 20-min open field (OF) trial, mean velocity (cm s^−1^), distance traveled (cm), and time spent in the center zone (%) were assessed at both time points, with a (E) representative trace shown of a vehicle-treated 5xFAD mouse. (I–L) Exploration time (s) and recognition index in the novel object recognition task (NORT) at 2 and 4 months of treatment. (M–P) Number of arm entries and percent alternation in the spontaneous alternation task (SAT) at 2 and 4 months of treatment. Data are presented as mean ± SEM and were analyzed using one-way ANOVAs with Tukey’s post-hoc test (*n* = 11–12). See also Figures S2B–S2E.

To assess whether GLP-1R agonist treatment altered behavioral outcomes in 5xFAD mice (Figure 2A), the open field (OF) test, novel object recognition task (NORT), and spontaneous alternation task (SAT) were conducted after 2 and 4 months of treatment. Notably, locomotion and anxiety-like behaviors were also assessed at baseline in an OF arena where 5xFAD mice were found to exhibit hyperactivity compared to WT mice with a significantly higher mean velocity and distance traveled in the 20-min trial (Figures S2B–S2D). Anxiety-like levels, measured as the percent time spent in the center zone, were comparable across genotypes at baseline, indicating this factor is likely not contributing to the observed hyperactivity (Figure S2E). Hyperactivity has been previously reported in this model, although at different ages.^23^^,^^24^^,^^25^ Despite this early genotype effect on locomotion, there were no significant genotype or drug effects on locomotion or anxiety-like behavior in the OF arena after 2 or 4 months of treatment (Figures 2B–2H).

The NORT and SAT are commonly used tasks in rodents to assess recognition memory and spatial working memory, respectively. WT vehicle mice performed in the expected range for cognitively intact animals in both tasks.^23^ At each assessed time point, there were no significant genotype or drug effects on object exploration time or recognition index in the NORT (Figures 2I–2L). Similarly, no significant effects were observed on the number of arm entries or percent alternation in the SAT (Figures 2M–2P). Overall, semaglutide and tirzepatide did not induce any nootropic effects in 5xFAD mice in these cognitive tasks.

### Effect of semaglutide and tirzepatide on whole-brain Aβ pathology in 5xFAD mice

**Figure 3:**
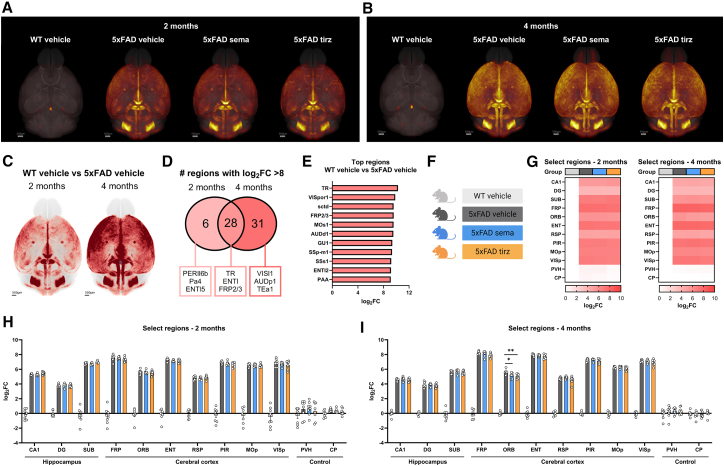
Effect of semaglutide and tirzepatide on whole-brain Aβ pathology in 5xFAD mice (A–I) (A and B) Representative images of dorsal overview of whole-brain 3D average Aβ plaque coverage, detected via an anti-Aβ_42_ antibody, in glow scale from WT or 5xFAD mice treated on alternate days with either vehicle, semaglutide (10 nmol kg^−1^), or tirzepatide (10 nmol kg^−1^) for 2 or 4 months. Scale bars, 500 μm. (C) Representative dorsal overview of whole-brain 3D Aβ plaque coverage of the difference between 5xFAD vehicle group vs. the WT vehicle group after 2 or 4 months of treatment. In all brain images, tissue autofluorescence is gray, and the scale bars represent 500 μm. (D) Venn diagram showing the number of brain regions with a log_2_fold change (FC) greater than 8, comparing WT vehicle to 5xFAD vehicle, in the 2- and 4-month cohorts. Examples of top regulated regions at each time point and shared between them are included below the Venn diagram. (E) Top regulated regions shared between the 2- and 4-month time points, presented as mean log_2_FC. (F–I) Aβ plaque coverage in preselected regions of interest, analyzed as the log_2_FC relative to the mean of the WT vehicle group, across all groups at both time points presented in (G) heatmaps or (H and I) bar graphs. For (H and I), all 5xFAD groups regardless of treatment had significant increases in Aβ vs. WT vehicle in all selected brain regions except control (statistical significance from one-way ANOVA not presented on graph). ∗*p* < 0.05 and ∗∗*p* < 0.01 vs. 5xFAD vehicle via one-way ANOVA with Tukey’s post-hoc test. Data are presented as mean ± SEM. Abbreviations for brain regions are defined in the methods. See also Figure S3.

Whole-brain 3D Aβ plaque deposition was assessed in mice in cohort 2 to determine whether 2 or 4 months of semaglutide or tirzepatide treatment altered this classical AD hallmark. As expected, WT vehicle mice had undetectable levels of Aβ at both time points (Figures 3A and 3B). First, an unbiased approach was taken to determine the top regulated regions between WT vehicle and 5xFAD vehicle groups. Already at 4 months of age (2-month treatment), there was widespread Aβ staining detected throughout the entire rostral to caudal axis of the 5xFAD brain (Figure 3C), with greater than 8-log_2_fold increases in 34 regions dominated by cortical areas such as the piriform, gustatory, entorhinal, motor, and somatosensory areas and the frontal pole (Figures 3D and S3A). Cortical-amygdalar and piriform-amygdalar areas were also top hits at this earlier time point (Figure S3A). Aβ plaque coverage was upregulated in 28 of these same regions at 6 months of age (4-month treatment), with 31 additional regions emerging as top hits including the auditory and visual cortices (Figures 3D, 3E, and S3B). This spatiotemporal spread of Aβ pathology throughout the brain mimics the known disease progression in this model which, maybe surprisingly, has also been found to translate well to human AD brain alterations.^26^

Next, the Aβ plaque deposition in preselected regions of interest known to be affected in AD were analyzed across groups (Figure 3F). After both 2 and 4 months of treatment, there were profound and consistent increases in Aβ coverage in all 5xFAD brains compared to WT vehicle, regardless of the treatment (Figures 3G–3I). This included hippocampal subregions such as region CA1 (CA1), the dentate gyrus (DG), and subiculum (SUB) and cortical regions including the frontal pole (FRP), orbital area (ORB), entorhinal area (ENT), retrosplenial area (RSP), piriform area (PIR), primary motor area (MOp), and primary visual area (VISp). Control regions including the paraventricular hypothalamic nucleus (PVH) and caudoputamen (CP) had non-significant changes in Aβ coverage (Figures 3H and 3I). Notably, the only significant drug effect was detected in the ORB where 4 months of semaglutide and tirzepatide treatment decreased Aβ plaque load (Figure 3I).

Finally, to validate the findings of this 3D analysis using an alternate method, 2D thioflavin S staining was conducted on brains from the same 2-month-treated mice. Regardless of treatment, all 5xFAD mice had significant increases in plaque load in the SUB compared to WT vehicle mice (Figures S3C–S3F). Incretin therapy also did not alter plaque compaction, indicated by no differences in average plaque diameter (Figure S3G). Overall, semaglutide and tirzepatide could not prevent the widespread progression of Aβ burden in this aggressive, amyloid-centric 5xFAD model.

### Effects of semaglutide and tirzepatide on microglia and astrocytes in 5xFAD mice

**Figure 4:**
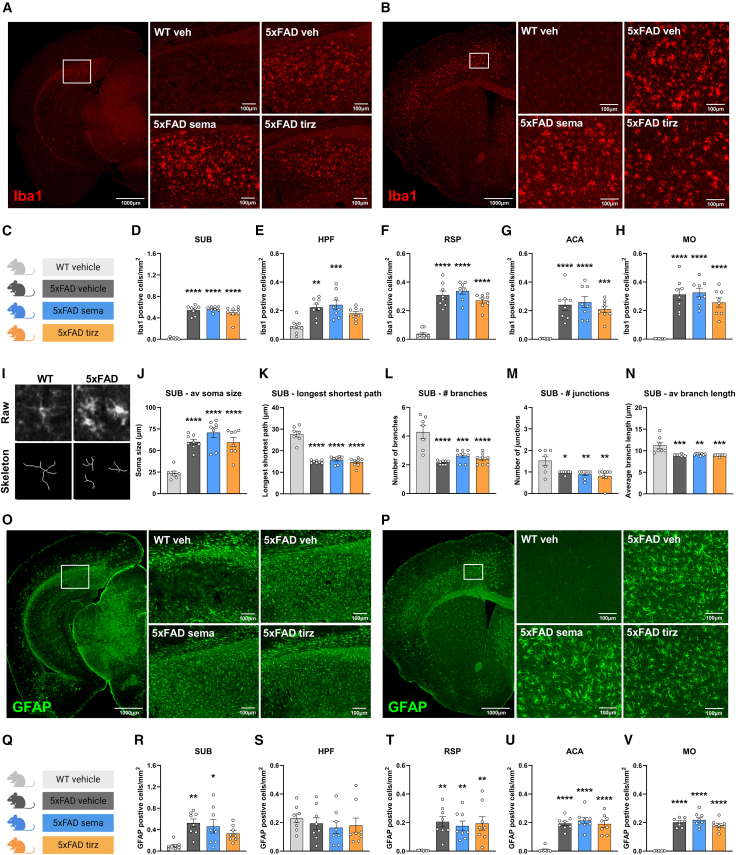
Effect of 2 months of semaglutide and tirzepatide treatment on microglia and astrocytes in 5xFAD mice (A–V) (A–C) Representative images of Iba1-positive microglia in the (A) hippocampus or (B) prefrontal cortex of WT or 5xFAD mice in cohort 2.1 treated on alternate days with either (C) vehicle, semaglutide (10 nmol kg^−1^), or tirzepatide (10 nmol kg^−1^) for 2 months. The half-brain sections displayed are from a representative 5xFAD vehicle mouse (scale bars, 1,000 μm), and the zoom box highlights the region the representative images shown are from (scale bars, 100 μm). (D–H) Quantification of the number of Iba1-positive cells per mm^2^ in the (D) subiculum (SUB), (E) hippocampal formation minus subiculum (HPF), (F) retrosplenial area (RSP), (G) anterior cingulate area (ACA), and (H) motor area (MO). Details on the section coordinates and regions of interest corresponding to the quantification in the bar graphs are found in Figure S6. (I) Examples of raw and skeletonized Iba1-positive cells from representative WT and 5xFAD mice. (J–N) Quantification of the morphology of Iba1-positive cells in the SUB including (J) average soma size (μm), (K) longest shortest path (μm), (L) number of branches, (M) number of junctions, and (N) average branch length (μm). (O–Q) Representative images of GFAP-positive astrocytes in the (O) hippocampus or (P) prefrontal cortex in the same mice. (R–V) Quantification of the number of GFAP-positive cells per mm^2^ in the (R) SUB, (S) HPF, (T) RSP, (U) ACA, and (V) MO. Data are presented as mean ± SEM. ∗*p* < 0.05, ∗∗*p* < 0.01, ∗∗∗*p* < 0.001, ∗∗∗∗*p* < 0.0001 vs. WT vehicle via one-way ANOVA with Tukey’s post-hoc test (n = 7–8). See also Figure S6.

**Figure 5:**
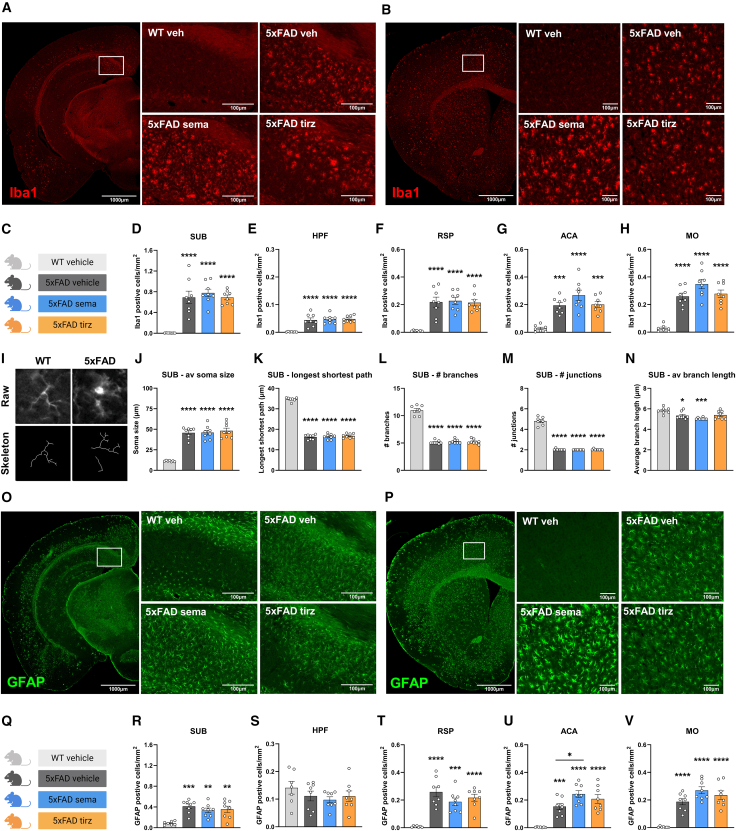
Effect of 4 months of semaglutide and tirzepatide treatment on microglia and astrocytes in 5xFAD mice (A–V) (A–C) Representative images of Iba1-positive microglia in the (A) hippocampus or (B) prefrontal cortex of WT or 5xFAD mice in cohort 2.2 treated on alternate days with either (C) vehicle, semaglutide (10 nmol kg^−1^), or tirzepatide (10 nmol kg^−1^) for 4 months. The half-brain sections displayed are from a representative 5xFAD vehicle mouse (scale bars, 1,000 μm), and the zoom box highlights the region the representative images shown are from (100 μm). (D–H) Quantification of the number of Iba1-positive cells per mm^2^ in the (D) subiculum (SUB), (E) hippocampal formation minus subiculum (HPF), (F) retrosplenial area (RSP), (G) anterior cingulate area (ACA), and (H) motor area (MO). Details on the section coordinates and regions of interest corresponding to the quantification in the bar graphs are found in Figure S6. (I) Example raw and skeletonized Iba1-positive cells from representative WT and 5xFAD mice. (J–N) Quantification of the morphology of Iba1-positive cells in the SUB including (J) average soma size (μm), (K) longest shortest path (μm), (L) number of branches, (M) number of junctions, and (N) average branch length (μm). (O–Q) Representative images of GFAP-positive astrocytes in the (O) hippocampus or (P) prefrontal cortex in the same mice. (R–V) Quantification of the number of GFAP-positive cells per mm^2^ in the (R) SUB, (S) HPF, (T) RSP, (U) ACA, and (V) MO. Data are presented as mean ± SEM. ∗*p* < 0.05, ∗∗*p* < 0.01, ∗∗∗*p* < 0.001, ∗∗∗∗*p* < 0.0001 vs. WT vehicle (unless otherwise specified) via one-way ANOVA with Tukey’s post-hoc test (n = 7–8). See also Figure S6.

Neuroinflammation is a hallmark of AD that is characterized by a spatiotemporal cascade in the activation state of resident brain immune cells such as microglia and astrocytes. To assess whether semaglutide and tirzepatide exert protective actions on this hallmark in 5xFAD mice, microglia and astrocyte activation was visualized and quantified via immunohistochemistry across multiple brain regions and time points (Figures S4A and S4B). At both 4 and 6 months of age (2 and 4 months of treatment), WT vehicle mice had very low levels of microgliosis or astrogliosis (Figures 4A–4V and 5A–5V). Although, notably astrocyte activation, measured via glial fibrillary acidic protein (GFAP) expression, was present throughout the hippocampal formation (HPF) in WT mice (Figures 4S and 5S).

At 2 months of treatment, there was a significantly greater number of Iba1-positive microglia (Figures 4A–4H) and GFAP-positive astrocytes (Figures 4O–4V) in 5xFAD vehicle-treated mice compared to WT vehicle-treated mice in all measured brain regions including the subiculum (SUB), HPF, retrosplenial area (RSP), anterior cingulate area (ACA), and motor area (MO), except for GFAP in the HPF. This activation pattern was maintained at 4 months of treatment (Figure 5) and concurs with the known progression of neuroinflammation in 5xFAD mice where the subiculum in the hippocampus is highly vulnerable at early stages, as well as deeper cortical layers connected to the limbic system.^27^^,^^28^

Neither semaglutide nor tirzepatide significantly altered microglia or astrocyte activation after 2 months of treatment in the measured regions of interest compared to 5xFAD vehicle controls (Figure 4). However, compared to WT vehicle mice, tirzepatide-treated 5xFAD mice did not have significantly different Iba1 expression in the HPF or GFAP expression in the SUB, suggesting the activation state of the cells have been somewhat dampened with tirzepatide treatment in these regions (Figures 4E and 4R). Four months of semaglutide or tirzepatide treatment also did not significantly alter the high load of Iba1-positive microglia (Figures 5A–5H) or GFAP-positive astrocytes (Figures 5O–5V) compared to 5xFAD vehicle mice in any region, except for the ACA where semaglutide further increased GFAP expression (Figure 5U).

To further examine the activation state of Iba1-positive microglia, morphological analysis was conducted (Figure S5). In all 5xFAD mice, regardless of treatment and at both time points, there was a significant increase in the average soma size and consistent decreases in the longest shortest path measured in the cell skeleton, the number of branches and junctions, and the average branch length of microglial cells in the SUB and ACA (Figures 4I–4N, 5I–5N, and S6). Together, these measures suggest a shift in microglial state in 5xFAD mice, from a homeostatic-like state with small soma and branching, to a more reactive or amoeboid-like state with decreased branch ramification and arborization. This supports the known dynamic state of microglia in 5xFAD mice where sustained activation of neuroinflammatory pathways promotes chronic microgliosis. Overall however, neither incretin therapy was able to convincingly alter the activation state of microglia or astrocytes after 2 or 4 months of treatment.

### Regulatory role of semaglutide and tirzepatide on gene expression of inflammatory and synaptic markers in 5xFAD mice

**Figure 6:**
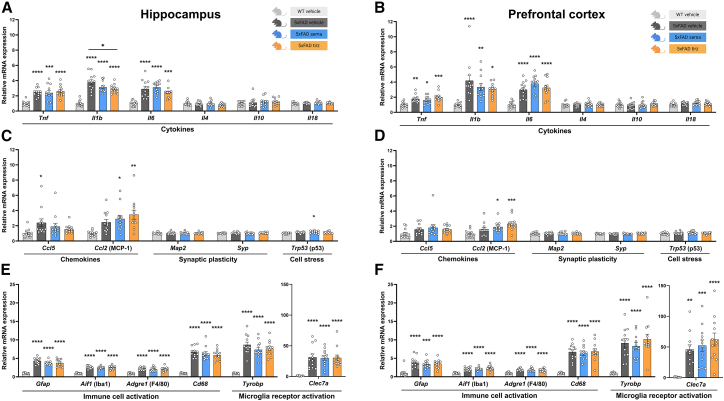
Regulatory role of semaglutide and tirzepatide on gene expression of inflammatory and synaptic markers in 5xFAD mice Brains were collected from female WT or 5xFAD mice in cohort 1 that were treated on alternate days with either vehicle, semaglutide (10 nmol kg^−1^), or tirzepatide (10 nmol kg^−1^) for 4 months. Quantitative PCR analysis of transcript levels of a range of (A and B) cytokines (*Tnf*, *Il1b*, *Il6*, *Il4*, *Il10*, and *Il18*); (C and D) chemokines (*Ccl5* and *Ccl2* [MCP-1]); and markers of synaptic plasticity (*Map2* and *Syp*) and cell stress (*Trp53* [p53]); (E and F) immune cell activation (*Gfap*, *Aif1* [Iba1], *Adgre1* [F4/80], and *Cd68*); and microglia receptor activation (*Tyrobp* and *Clec7a*). Data are presented for the (A, C, and E) hippocampus and (B, D, and F) prefrontal cortex as mRNA expression relative to the mean of the WT vehicle group. Data are presented as mean ± SEM. ∗*p* < 0.05, ∗∗*p* < 0.01, ∗∗∗*p* < 0.001, ∗∗∗∗*p* < 0.0001 vs. WT vehicle (unless otherwise specified) via one-way ANOVAs with Tukey’s post-hoc test (*n* = 11–12).

GLP-1R agonists are thought to mediate their central protective actions by modulating inflammatory and synaptic pathways.^4^ Therefore, the gene expression of a range of inflammatory and synaptic markers was assessed in semaglutide- and tirzepatide-treated 5xFAD mice at the 4-month time point via qPCR. Compared to WT vehicle-treated mice, 5xFAD vehicle-treated mice had significantly increased levels of inflammatory markers in both the hippocampus and prefrontal cortex (Figures 6A–6F). For example, the pro-inflammatory cytokines, *Tnf*, *Il1b*, and *Il6*, increased 2- to 4-fold in 5xFAD mice (Figures 6A and 6B). This change is likely due to an increase in microglial cell number. Cell activation markers for astrocytes (*Gfap*) and microglia/resident macrophages (*Aif1* [Iba1], *Cd68*, *Adgre1* [F4/80]) were consistently increased, along with the microglial pattern recognition receptor *Clec7a* and the microglial adaptor protein *Tyrobp* (Figures 6E and 6F). The chemokine, *Ccl5*, significantly increased only in the hippocampus of 5xFAD vehicle-treated mice (Figure 6C).

Chronic treatment with semaglutide or tirzepatide did not alter the gene expression of any of the tested inflammatory markers compared to the 5xFAD vehicle group, except for a significant decrease in *Il1b* in the hippocampus with tirzepatide treatment (Figure 6A). Notably, relative to the WT vehicle group, both incretin receptor agonists increased the gene expression of the chemokine *Ccl2* (MCP-1) in the hippocampus and prefrontal cortex (Figures 6C and 6D). Interestingly, while high plasma MCP-1 levels are associated with increased symptom severity in patients with AD,^29^ there is also evidence to support an improvement in Aβ plaque clearance via CCR2-dependent recruitment of peripheral monocytes at early AD disease stages.^30^^,^^31^ Lastly, the cell stress regulator, *Trp53* (p53), increased only in the hippocampus with semaglutide treatment (Figure 6C). There were no significant genotype or drug effects on the cytokines, *Il4*, *Il10*, or *Il18*, or the synaptic markers, *Map2* or *Syp* (synaptophysin) (Figures 6A–6D).

### Effects of semaglutide and tirzepatide on neuroinflammation in LPS-treated mice

Overall, chronic treatment of semaglutide or tirzepatide did not convincingly alter the progression of AD-like pathology in the aggressive, amyloid centric, transgenic 5xFAD mouse. To test the preventative protective actions of these drugs in an alternate AD mouse model with a different pathogenesis and pathological trajectory, the LPS model of neuroinflammation was used. It is well established that peripheral administration of low doses of the bacterial endotoxin LPS elicits a robust systemic inflammatory response including widespread inflammation in the CNS.^32^^,^^33^^,^^34^

**Figure 7:**
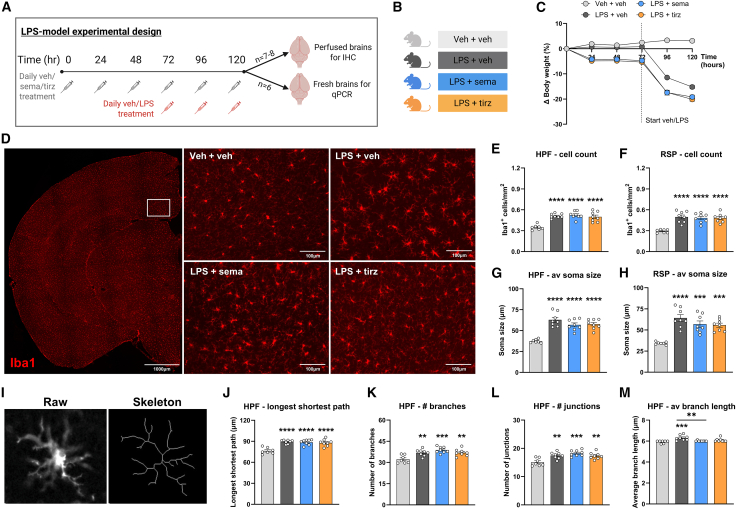
Effect of semaglutide and tirzepatide treatment on microglia in LPS-treated mice (A and B) Schematics of experimental design and groups in LPS studies. Two separate cohorts of 10-week-old male C57BL/6J mice were treated daily for 3 days with either vehicle (veh), semaglutide (10 nmol kg^−1^; sema), or tirzepatide (10 nmol kg^−1^; tirz) followed by another 3 days of drug and either vehicle (veh) or lipopolysaccharide (250 μg kg^−1^; LPS). Mice in one cohort were perfused and brains used for immunohistochemistry experiments. Fresh frozen brains from mice in the other cohort were used for quantitative PCR experiments. (C) Daily percent change in body weight from baseline in mice across both cohorts (*n* = 13–14). (D) Representative images of microglia (stained with Iba1) in sections of the hippocampus of each group. The half-brain section displayed is from a representative LPS + veh mouse (scale bars, 1,000 μm), and the zoom box highlights the region the representative images shown are from (scale bars, 100 μm). (E and F) Quantification of the number of Iba1-positive cells per mm^2^ in the (E) hippocampal formation (HPF) and (F) retrosplenial area (RSP). (G and H) Quantification of the average soma size (μm) of Iba1-positive cells in the (G) HPF and (H) RSP. (I) Example raw and skeletonized images of a single microglial cell. (J–M) Quantification of the morphology of Iba1-positive cells in the HPF including (J) longest shortest path (μm), (K) number of branches, (L) number of junctions, and (M) average branch length (μm). Data are presented as mean ± SEM. ∗∗*p* < 0.01, ∗∗∗*p* < 0.001, ∗∗∗∗*p* < 0.0001 vs. veh + veh (unless otherwise specified) via one-way ANOVA with Tukey’s post-hoc test (*n* = 7–8). See also Figure S7.

In our study, 10-week-old male C57BL/6J mice were pre-treated for 3 days with daily semaglutide or tirzepatide injection (Figures 7A and 7B). This was followed by 3 days of daily co-treatment of the incretin mimetic and a low dose of LPS (250 μg kg^−1^, intraperitoneal [i.p.]). Semaglutide and tirzepatide treatment resulted in an initial drop in body weight relative to baseline (4.7% ± 0.4% for semaglutide and 5.3% ± 0.6% for tirzepatide; Figure 7C). Subsequent LPS administration potently reduced body weight by 15.1% ± 2.7% in all LPS-treated mice, with a total 19.2% ± 0.8% decrease in LPS + semaglutide-treated mice and a 20.1% ± 0.7% decrease in LPS + tirzepatide-treated mice at endpoint relative to baseline (Figure 7C). Notably, LPS also induced acute sickness-like behavior including decreased locomotor activity and a lack of response to handling, as per previous reports in this model.^32^^,^^34^

Neuroinflammatory processes in drug-treated mice were examined at endpoint by assessing microglial cell state using immunohistochemistry and the gene expression of various inflammatory markers via qPCR. First, there was a significant increase in the number of Iba1-positive cells in the hippocampal formation (HPF) and retrosplenial area (RSP) in LPS + vehicle-treated mice compared to vehicle + vehicle-treated mice (Figures 7D–7F). This was not altered with pre-treatment of either semaglutide or tirzepatide (Figures 7D–7F). Morphological analysis of Iba1-positive microglia in the HPF also revealed that LPS stimulation significantly increased the average soma size, the longest shortest path measured in the cell skeleton, the number of branches and junctions, and, to a lesser extent, the average branch length (Figures 7G–7M and S5). These morphological changes were not altered by semaglutide or tirzepatide. Together, these measures suggest a shift in microglial state in all LPS-treated mice regardless of group, from a homeostatic-like state with a small soma and some branching, to a hyper-ramified-like state with increased branch ramification and arborization. These findings align with the timing of brain collection at 4 h after the last LPS injection and suggest the cells are in a heightened surveillance state in response to the acute LPS insult.

The gene expression of inflammatory markers was also measured in drug-treated mice (Figure S7). There were robust, albeit highly variable, increases in the cytokines *Tnf*, *Il1b*, *Il10*, and *Il6*, and the chemokine *Ccl2* in the hippocampus and prefrontal cortex of LPS + vehicle mice compared to vehicle + vehicle mice (Figures S7A–S7D). Similar increases in the immune cell activation markers *Gfap*, *Aif1* (Iba1), and *Adgre1* (F4/80), as well as an upregulation in the LPS receptor *Tlr4* were also observed in LPS-treated groups (Figures S7B and S7D). There were no significant alterations in these upregulated inflammatory markers with semaglutide or tirzepatide treatment. However, there are subtle indications of lowered pro-inflammatory cytokines with tirzepatide pre-treatment in both regions. There were no detectable LPS-induced differences between groups in the markers *Il4*, *Il18*, and *Glp1r* (Figures S7B and S7D). Although, incretin treatment significantly lowered *Il4* gene expression in the prefrontal cortex (Figure S7C). Lastly, it is worth noting that Aβ plaque staining with both an anti-Aβ_42_ antibody and thioflavin S was attempted in LPS-treated brain sections, although no plaques were detected with either technique in this model. This is congruent with inconsistent reports in previous studies.^33^^,^^34^

## Discussion

There is an increasing body of evidence supporting pleiotropic protective actions of the GLP-1R agonist semaglutide and the GLP-1R/GIPR co-agonist tirzepatide across multiple organ systems, including the CNS. However, mixed findings from preclinical AD models and early clinical studies leave open the question of whether these drugs can meaningfully influence AD progression. We therefore set out to characterize their effects in the 5xFAD mouse, a widely used transgenic model characterized by early and substantial amyloid pathology. Additionally, results were translated into an alternate mouse model, the LPS model of neuroinflammation, which has a different disease pathogenesis and trajectory not dependent on amyloid expression. Our experimental design in both models aimed to maximize the likelihood of detecting efficacy. Instead of attempting to reverse established pathology, we implemented a preventive paradigm beginning in young animals, used doses known to produce robust metabolic effects in rodents, and maintained treatment for long durations to ensure continuous target engagement. This approach provided a stringent test of whether semaglutide or tirzepatide could alter disease trajectory under conditions in which their systemic pharmacology is reliably active.

Despite this design, neither semaglutide nor tirzepatide convincingly altered the progression of AD-like pathology in either model. This was particularly evident in the whole-brain analysis of Aβ plaque coverage in 5xFAD mice where no significant drug effects were observed, except in the orbital area after 4 months of treatment. Similarly, neither semaglutide nor tirzepatide significantly altered the activation of microglia or astrocytes or the gene expression of key cytokines, chemokines, and cell activation markers in the hippocampus or prefrontal cortex of 5xFAD or LPS-treated mice. Together, these results suggest that these two clinically relevant compounds do not prevent the progression of pathological hallmarks of AD including Aβ plaques and neuroinflammation, in these two distinct mouse models.

Given the absence of detectable effects on neuropathology, it is unsurprising that the drugs did not alter performance in behavioral assays in 5xFAD mice. It is worth noting however that there was also no genotype effect detected in either memory task, suggesting that the robust pathology seen in the brain had not yet translated into measurable behavioral deficits in these tasks. This absence of measurable memory impairment is consistent with prior reports showing that 5xFAD mice of a similar age can display substantial pathology without robust deficits in these behavioral tasks.^25^^,^^35^ Future studies with an experimental design that prioritizes comprehensive screening of hippocampus-dependent behavioral tasks as the primary endpoint are needed to conclusively determine the effect of these compounds on mouse memory and learning.

While not considered a classic AD hallmark, systemic metabolic dysfunction has been reported in both patients with AD and in the 5xFAD mouse.^22^^,^^36^ For example, 5xFAD mice have been suggested to exhibit a state of negative energy balance, characterized by reduced food intake, hypothalamic dysfunction despite the absence of detectable Aβ plaques, and neuroendocrine abnormalities including low circulating insulin and impaired insulin signaling.^22^ This is concurrent with the findings of our study where 5xFAD vehicle-treated mice were leaner after 3 months of age, were glucose intolerant, and had significantly lower levels of plasma insulin. Interestingly, both semaglutide and tirzepatide improved glucose tolerance without affecting plasma insulin levels. This may suggest that semaglutide and tirzepatide improve glucose tolerance in 5xFAD mice through mechanisms independent of increased insulin secretion. Given the low circulating insulin levels in vehicle-treated 5xFAD mice, this improvement could not only reflect preserved or enhanced insulin sensitivity but also involve insulin-independent pathways such as reduced hepatic glucose output or altered autonomic regulation of glucose metabolism. In any case, the incretin agonists normalize aspects of glucose metabolism without correcting the underlying defect in insulin secretion. Notably, semaglutide- and tirzepatide-treated 5xFAD mice had lower plasma triglyceride levels, indicating that peripheral lipid metabolism remains responsive to incretin agonism even in the context of the altered metabolic state of this model.

A significant strength of the current study lies in the use of two AD mouse models with completely distinct mechanisms driving disease pathology. All downstream pathological features present in the transgenic 5xFAD model is driven by the aggressive and widespread deposition of Aβ plaques. In contrast, neuropathology observed in the LPS model relies on the sustained activation of innate inflammatory signals triggered by the highly potent bacterial endotoxin. While both models result in robust neuroinflammation, the distinct underlying driving mechanisms result in different pathological signatures of brain immune cells. This is clearly exemplified by the divergent morphological states of microglia observed in this study, where microglia in 5xFAD mice had a reactive or amoeboid-like state, compared to the highly ramified-like state in LPS-treated mice. The fact that neither semaglutide nor tirzepatide altered microglia morphology in either model strengthens our conclusion that these compounds had no measurable protective effects. Notably, it would be informative in future studies to examine the effects of these compounds in the CNS of healthy WT mice, to explore possible pathology-independent mechanisms of action on healthy neuron or glial cell biology.

There are a number of possible, likely interconnected, factors that could have contributed to the lack of convincing neuroprotective actions of semaglutide and tirzepatide in 5xFAD mice. The 5xFAD mouse exhibits early-onset, high-burden, amyloid-driven pathology, which narrows the therapeutic window and accelerates neurons toward a state where aggregate formation outpaces clearance.^37^ Once this trajectory is established, interventions that do not directly modulate amyloid kinetics might have limited room to alter disease course. This stands in contrast to passive anti-Aβ immunotherapies, whose mechanism closely aligns with the dominant pathology in this model. In contrast, incretin-based therapies act through broader metabolic and inflammatory pathways rather than directly on Aβ production or clearance. Their putative neuroprotective actions span modulation of neuroinflammation, support of cellular energy metabolism, and changes in synaptic function, but these benefits might only be relevant when pathology remains coupled to metabolic and immune state.^4^ In a largely amyloid-centric model with rapid onset and a lesser contribution from these broader physiological drivers, such indirect mechanisms appear to be insufficient in producing measurable shifts in pathology.

Despite the reported central anti-inflammatory properties of GLP-1R agonism, neither semaglutide nor tirzepatide convincingly altered inflammatory markers in LPS-treated mice. However, hints at subtle reductions in cytokine and chemokine gene expression with tirzepatide suggest the addition of GIPR agonism may provide superior central anti-inflammatory actions to GLP-1R agonism alone. Further testing of the effects of tirzepatide on neuroinflammatory pathways is warranted, particularly in human cells and tissues, given that tirzepatide has a relatively poor efficacy at the mouse GIPR, making mice poorly suited to fully capture the GIPR-dependent pharmacology from tirzepatide.

It is also important to note that although incretin therapy was initiated at an early age in the 5xFAD study, AD-related pathological processes were likely already underway. As such, the study may be best viewed as an early-intervention paradigm rather than a purely preventative one. Epidemiological studies provide strong support for the preventative actions of semaglutide in reducing dementia risk.^6^ However, these protective actions are yet to be translated into a population in which disease pathology is already present such as in the EVOKE and EVOKE+ phase III trials where patients were amyloid-positive despite being at early AD stages.^7^ Our study design has similarities with that of the EVOKE trials, with treatment onset at an early disease stage where disease trajectory is already established. Together, the clinical and preclinical evidence suggests that any potential protective actions of these compounds is likely to lie in disease prevention when pathology has not yet begun or in settings where the primary pathological features such as Aβ plaques have been removed via other means.

Finally, long-acting incretin mimetics reach the brain only modestly and appear to access primarily circumventricular and hypothalamic regions, with slow or minimal penetration into deeper limbic structures such as the hippocampus. Although receptor expression in these regions is well documented, current drugs may not achieve levels sufficient for sustained engagement.^4^ Importantly, the regions in which pathology accumulates most rapidly in 5xFAD mice, and also in patients with AD, coincide with those that current incretin mimetics reach only minimally, further limiting the likelihood of observing robust efficacy in this model. This limitation also underscores the potential negative clinical results in the EVOKE trials.^7^ Interestingly, treatment with the earlier generation GLP-1R agonist liraglutide was recently reported to improve cognitive outcomes measured via the Assessment Scale-Executive domain (ADAS-Exec) in patients with mild-to-moderate AD in a phase IIb clinical trial.^38^ This could be explained, at least in part, by the observation that earlier generation incretin mimetics have, to varying extents, improved brain permeability over a prolonged period.^39^^,^^40^ This point also contributes to the disparity in results from preclinical studies testing incretin mimetics in AD models. Previous studies testing exenatide and liraglutide report positive actions in improving AD pathology while more recent testing of semaglutide and tirzepatide, including ours, observe no significant drug effects.^8^^,^^9^^,^^10^^,^^11^^,^^12^^,^^14^^,^^18^^,^^21^ Differences in the experimental setup including the model used, prevention vs. reversal design, and treatment duration likely also contribute to the mixed reports. The main strengths of our study, which aimed to maximize the detection of potential drug effects, include the preventative design, long treatment duration, and use of two distinct yet complementary models, in addition to the molar-matched doses of clinically relevant compounds. This positions our study as a key reference point to guide the design of future preclinical investigations testing next-generation incretin mimetics that have improved pharmacokinetics and brain access.

Taken together, our findings indicate that chronic semaglutide or tirzepatide treatment, even when initiated before overt pathology and delivered for a prolonged period, does not slow the progression of amyloid deposition or neuroinflammatory activation in the 5xFAD mouse. The modest metabolic benefits we observed, including improved glucose tolerance and reduced circulating triglycerides, did not translate into detectable protection against early AD-like neuropathology in this model. The recently announced EVOKE and EVOKE+ results, which similarly reported no clinical benefit of semaglutide despite biomarker engagement, reinforce the notion that metabolic or indirect anti-inflammatory modulation may be insufficient to counter rapid amyloid-driven neurodegeneration once pathological trajectories are firmly established. However, subtle indications that tirzepatide modulates inflammatory pathways in LPS-treated mice suggests the addition of GIPR agonism may provide protective effects beyond targeting GLP-1R agonism alone.

Rather than closing the door on incretin biology in AD, these observations point to the possibility that next-generation incretin-based agents, with differentiated receptor engagement, optimized pharmacokinetics, or improved CNS penetrance, together with models that better reflect the slower, multifactorial evolution of human late-onset AD, may be required to reveal any potential disease-modifying effects. Within such contexts, contributions from metabolic and inflammatory mechanisms remain a plausible component of the broader therapeutic landscape of AD.

### Limitations of the study

While our experimental design in both models aimed to maximize the likelihood of detecting drug efficacy, it is possible that higher doses would provide more potent modulatory effects on AD neuropathology. Additionally, there are a number of limitations to the mouse models used in this study, as discussed in detail earlier, including the aggressiveness of the amyloid pathology in 5xFAD mice. The lack of robust neurofibrillary tangle pathology, another hallmark of AD, in these models also presents a significant limitation. Extrapolation of the findings of this study between sexes is also not possible, as female 5xFAD mice and LPS-treated male C57BL/6J mice were used. Future studies should test the potential protective actions of semaglutide and tirzepatide on memory and learning in rodent models with more robust behavioral deficits. Expanded behavioral assays that depend on the native behavior of the animal would also be informative, including paradigms such as nesting and the marble burying test, as well as more sensitive cognitive assays such as the Morris water maze. Lastly, functional studies on the effects of incretin therapy on neuronal circuits in the context of AD would greatly strengthen the mechanistic interpretation of this work.

## Resource availability

### Lead contact

Further information and requests for resources and reagents should be directed to and will be fulfilled by the lead contact, Christoffer Clemmensen (chc@sund.ku.dk).

### Materials availability

This study did not generate new unique reagents.

### Data and code availability

• The datasets generated and analyzed during this study are available from the corresponding author upon request.
• This paper does not report original code.
• Any additional information required to reanalyze the data reported in this work is available from the lead contact upon request.

## Acknowledgments

We thank Jacob Hecksher-Sørensen, Urmas Roostalu, Micaela Roque, and others at Gubra for their contributions to the 3D imaging studies. We acknowledge the Core Facility for Integrated BioImaging, Faculty of Health and Medical Sciences, University of Copenhagen. We would also like to acknowledge past and present members of the Clemmensen group including Stina Börchers, Federico Picciau, Mathilde Balle, and Emilie Springborg for their contributions to the LPS experiments. This work was supported by the Independent Research Fund Denmark (grant no. 4285-00413A). Financial support to C.C. was provided through the 10.13039/501100009708Novo Nordisk Foundation (NNF22OC0073778) and the 10.13039/501100000781European Research Council under the European Union’s 10.13039/100018693Horizon Europe research and innovation program (grant agreement no. 101170678, PDC-MAIN). The 10.13039/501100011747Novo Nordisk Foundation Center for Basic Metabolic Research is an independent Research Center, based at the 10.13039/501100001734University of Copenhagen, Denmark, and partially funded by an unconditional donation from the 10.13039/501100009708Novo Nordisk Foundation (www.cbmr.ku.dk) (NNF23SA0084103).

## Author contributions

A.V. and C.C. conceptualized the project and wrote the manuscript. A.V., S.A.O., E.C.H.L., M.A.M., and C.S. were involved in experimental methodology. A.V. analyzed all data. C.C. acquired funding and supervised the project. All authors contributed to the final reading and editing of the manuscript.

## Declaration of interests

C.C. is a co-founder of Ousia Pharma and Heureka Therapeutics, biotechnology companies developing therapeutics for the treatment of cardiometabolic diseases.

## STAR★Methods

### Key resources table

**Table:** 

REAGENT or RESOURCE	SOURCE	IDENTIFIER
**Antibodies**		

Rabbit anti-human Amyloid Beta	IBL	Cat#18584; RRID: AB_10705431
Donkey anti-rabbit Cy5	Jackson ImmunoResearch	Cat#711-175-152; RRID: AB_2340607
Goat anti-Iba1	Abcam	Cat#ab5076; RRID: AB_2224402
Rabbit anti-GFAP	Abcam	Cat#ab68428; RRID: AB_1209224
Donkey anti-goat 568 Alexa Fluor	Invitrogen	Cat#A-11057; RRID: AB_2534104
Donkey anti-rabbit 488 Alexa Fluor	Invitrogen	Cat#A-21206; RRID: AB_2535792

**Chemicals, peptides, and recombinant proteins**		

Dithiothreitol	Thermo Fisher	18080–044
FS buffer	Thermo Fisher	18080–044
LPS-EB (LPS from E.coli O111:B4)	Invivogen	tlrl-eblps; Lot# 59694501
PrecisionPLUS qPCR Master Mix with SYBRgreen	Primer Design	Z-PPLUS-SY-1ML
RNaseOUT	Thermo Fisher	10777019
Semaglutide	MedChemExpress	HY-114118
SuperScript III	Thermo Fisher	18080–044
Thioflavin S	Sigma-Aldrich	T1892
Tirzepatide	MedChemExpress	HY-P1731
Tissue-Tek OCT compound	Sakura	4583
TRIzol reagent (QIAzol Lysis Reagent)	Qiagen	79306
Vectashield hardset mounting medium with DAPI	Vector Labs	H-1500-10

**Critical commercial assays**		

iScript cDNA synthesis kit	BioRad	1708891
Mouse Insulin ELISA kit	Crystal Chem	90080
Non-esterified fatty acids (NEFA)-HR assay kit	Wako Fujifilm	434–91795
Total Cholesterol LiquiColor kit	Stanbio	1010–225
Triglyceride LiquiColor kit	Stanbio	2100–225
QIAwave RNA Mini Kit	Qiagen	74534

**Experimental models: Organisms/strains**		

Mouse: Tg(APPSwF1Lon, PSEN1∗M146L∗L286V)6799Vas/Mmjax	The Jackson Laboratory	RRID: MMRRC_034840-JAX
Mouse: C57BL/6JRj	Janvier Laboratories	RRID: IMSR_RJ:C57BL-6JRJ

**Oligonucleotides**		

dNTP	Thermo Fisher	R0192
Primers for qPCR, see Table S1	This paper	N/A
Random primers	Sigma-Aldrich	11034731001

**Software and algorithms**		

Ethovision XT	Noldus	https://noldus.com/ethovision-xt
Prism	GraphPad	https://www.graphpad.com/
ImageJ	Schneider et al.^41^	https://imagej.net/ij/
Imaris	Oxford Instruments	https://imaris.oxinst.com/

### Experimental model

#### Animals

Age-matched female 5xFAD non-carrier wild-type (WT) (*n* = 28) and hemizygous mice (*n* = 84), generated on a B6SJLF1 background, were obtained from Jackson Laboratories (Tg(APPSwF1Lon, PSEN1∗M146L∗L286V)6799Vas/Mmjax, 034840-JAX). All mice inherited the transgene paternally. It is also worth mentioning that only female mice were used in this 5xFAD study due to their more rapid and aggressive phenotype. Therefore, findings across sexes should be extrapolated with caution. For LPS experiments, 10-week-old male C57BL/6JRj mice were obtained from Janvier Laboratories. Female mice were group-housed (3–4 per cage) and male mice were double-housed (2 per cage) at 21°C–23°C and 33–35% humidity on a 12 h–12 h light-dark cycle (06:00-18:00) with *ad libitum* access to water and regular diet (Altromin 1324, Brogaarden). All animal experiments were conducted according to international principles of animal care and under the approval of the Danish Ethical Committee for Animal Research and the Danish Animal Experimentation Inspectorate (License numbers: 2023-15-0201-01442, 2023-15-0201-01530).

#### Experimental design – 5xFAD study

Mice were randomly divided into four experimental groups based on genotype and treatment: vehicle-treated WT, vehicle-treated 5xFAD, semaglutide-treated 5xFAD, and tirzepatide-treated 5xFAD (*n* = 28 per group) (see Figure S1). Body weight was measured weekly throughout the treatment period, and twice during the first week to monitor potential acute treatment responses.

To investigate both behavioral and metabolic outcomes and reduce stress-induced responses, the animals were divided into two experimental cohorts (Figure S1). Cohort 1 (*n* = 12 per group) were treated for 4 months (16 weeks) and underwent behavioral testing at baseline and after 2 and 4 months of treatment. In parallel, cohort 2 underwent metabolic phenotyping including fortnightly blood glucose measurements, and compound and glucose tolerance testing. This cohort was further sub-divided into cohort 2.1 (*n* = 8 per group) which were treated for 2 months (8 weeks), and cohort 2.2 (*n* = 8 per group) which were treated for 4 months (16 weeks).

Mice in both cohorts began treatment at 2 months of age and received subcutaneous injections on alternate days between 09:00 and 11:00 of either vehicle (8 mM PBS/240 mM propylene glycol, pH 8.2) or molar-matched doses (10 nmol kg^−1^) of semaglutide or tirzepatide. All compounds were prepared as 1 mg mL^−1^ stock solutions and diluted to the final working concentration every second week in accordance with the known stability of the drugs.

#### Experimental design – LPS study

Ten-week-old male C57BL/6JRj mice were randomly divided into four experimental groups (n = 6–8): vehicle + vehicle, LPS + vehicle, LPS + semaglutide, and LPS + tirzepatide. Mice were pre-treated for 3 days with daily subcutaneous injections between 09:00 and 11:00 of either vehicle (8 mM PBS/240 mM propylene glycol, pH 8.2) or molar-matched doses (10 nmol kg^−1^) of semaglutide or tirzepatide. Following the 3 days of pre-treatment, mice were injected daily for another 3 days with vehicle/semaglutide/tirzepatide as well as an intraperitoneal injection of either vehicle (saline) or LPS (250 μg kg^−1^). The body weight and clinical severity score of each mouse was recorded daily before injection.

### Method details

#### Compound tolerance test

A compound tolerance test (CTT) was performed on cohort 2.1 in the 5xFAD study after 14 weeks of treatment (at 22 weeks of age) to assess acute changes in blood glucose in response to compound administration. The mice were not fasted prior to testing. Tail vein blood glucose was assessed using a glucometer at 0, 0.5-, 1-, 2- and 4-h after subcutaneous injection of the compound.

#### Glucose tolerance test

A glucose tolerance test (GTT) was performed on cohort 2.1 in the 5xFAD study after 15 weeks of treatment (at 23 weeks of age). Mice were acclimatized to the testing room and fasted for 4 h before being challenged by an intraperitoneal injection of glucose (2 mg kg^−1^) dissolved in isotonic saline. Tail vein blood glucose was assessed using a glucometer at 0, 15-, 30-, 60- and 120-min post-glucose injection.

#### Behavioral studies

Cohort 1 (*n* = 12) in the 5xFAD study underwent behavioral testing at baseline (only open field), and after 2- and 4-month of treatment. For all behavioral studies mice were acclimatized to the behavior room for at least 4 days prior to testing under the same housing conditions as outlined previously. Before baseline testing, mice were also handled twice daily for a week by the same two experimenters to reduce stress induced by handling during the behavioral tasks. Behavioral testing was conducted on days between injections during treatment and in the light phase. To reduce stress, wood chip bedding from the home cage was sprinkled into the arena before each trial. The arenas were thoroughly cleaned with 70% ethanol between trials. All trials were recorded and analyzed using Ethovision XT video tracking software (Noldus).

#### Open field test

To assess locomotion and anxiety-like behavior, mice were placed in an open field (OF) arena (50 cm^2^) with dimmable LED lighting and allowed to freely explore for 20 min. The mean velocity (cm s^−1^), distance traveled (cm), and time spent in the center zone (%) were analyzed for each mouse at baseline, and after 2- and 4- months of treatment.

#### Novel object recognition task

The novel object recognition task (NORT) was conducted on mice after 2- and 4-month of treatment to assess recognition memory. The OF task, conducted in the days immediately before, was used as habituation to the OF arena. In trial 1 of the NORT (familiarization phase), mice were placed in the OF arena containing two identical objects (either pyramids or domes) evenly spaced in opposing corners of the arena for 10 min. To satisfy inclusion in the experiment, the time the mouse spent exploring each individual object in the familiarization phase, quantified as the time the nose of the mouse was within a 2 cm radius of the object, needed to be greater than 15 s. After a 1-h inter-trial interval, mice were placed back into the same arena for trial 2 (testing phase) where one familiar object was replaced by a novel object. The location and type of object used as the novel object was randomized across groups. Preference for the novel object was calculated as a recognition index, defined as the time spent exploring the novel object divided by the total exploration time of both objects.

#### Spontaneous alternation task

To assess spatial working memory, the spontaneous alternation task (SAT) was conducted on 2- and 4-month-treated mice. Mice were placed in the center of a Y maze (30 cm long arms) and allowed to freely explore for 7 min. Visual cues were placed on the walls in the behavior room above each arm to provide spatial orientation. The sequence and number of arm entries, based on the center-point of the mouse, were recorded and an alternation score (%) was calculated.

#### Collection of blood and tissue samples

Mice in cohort 1 of the 5xFAD study were euthanized after 4 months of treatment via decapitation. Trunk blood was immediately collected in EDTA-coated microvette tubes and centrifuged at 14,000 rpm for 15 min at 4°C to isolate plasma, which was stored at −70°C until later analysis. Whole fresh brains were dissected, snap frozen and stored at −70°C for later biochemical analysis. To isolate the regions of interest from the whole frozen brains, thick sections were cut using a brain matrix and the regions of interest were punched out using a 1 mm Miltex Biopsy Punch. This process was conducted in a cryostat to ensure the tissue remained frozen.

Mice in cohort 2.1 and 2.2 of the 5xFAD study were euthanized after 2- and 4-month, respectively, via cardiac perfusion. Mice were first anesthetized with isoflurane followed by 2 min of perfusion with heparinized PBS and 5 min of perfusion with 10% formalin. Whole fixed brains were dissected and stored in 10% formalin overnight. The following day, the fixed brains were washed 3 times with PBS and then stored in 0.02% sodium azide/PBS until 3D whole brain imaging.

For immunohistochemistry experiments in the LPS study, mice were anesthetized with pentobarbital/lidocaine and perfused for 4 min with PBS. Whole brains were dissected and placed in 10% formalin. After overnight incubation, brains were prepared for 2D staining as described below.

For gene expression experiments in the LPS study, mice were euthanized via decapitation and whole fresh brains were collected. Regions of interest including the hippocampus and prefrontal cortex were immediately microdissected, snap frozen and stored at −70°C.

#### Plasma biomarker assays

Plasma from mice in cohort 1 in the 5xFAD study were analyzed for different metabolic biomarkers. It is worth noting that while absolute lipid values can vary somewhat depending on whether serum or plasma is used, experimental groups were processed and analyzed in parallel allowing for accurate comparison across groups. Plasma insulin levels were quantified using the Crystal Chem Ultra Sensitive Mouse Insulin ELISA kit (Crystal Chem, 90080). Plasma total cholesterol (Stanbio, 1010–225), triglycerides (Stanbio, 2100–225), and non-esterified fatty acids (NEFA) (Wako Fujifilm, 434–91795) were quantified using enzymatic kits according to the manufacturer’s protocols.

#### 3D whole brain Aβ staining and analysis

Amyloid-beta coverage in whole perfusion-fixed brains from mice in cohort 2 of the 5xFAD study was assessed using 3D light sheet fluorescence microscopy. These experiments were conducted at Gubra (Hørsholm, Denmark). Brains were prepared, immunolabelled and cleared according to the in-house optimized iDISCO+ protocol. The primary antibody used in this study was rabbit anti-human Amyloid Beta (IBL, 18584, diluted 1:100) and the secondary antibody was donkey anti-rabbit Cy5 (Jackson ImmunoResearch, 711-175-152). Notably, the staining intensity was strongest in the outer cortical layers, with a gradual reduction toward deeper structures. This spatial pattern was consistent across all mice. While this limits direct comparisons between regions, it still permits reliable group comparisons within each region.

Brain samples were imaged using an LCS SPIM microscope with 4X C objective. ECi was used as a clearing agent during acquisition of data. Imaris/Aivia software was used for 3D visualization of the data. Customized in-house developed software was used for all image analysis with Allen’s Brain Atlas used as the reference atlas. All data is presented as a log_2_fold change (FC) relative to the mean of the WT vehicle group.

Following the 3D Aβ imaging, cleared brains were rehydrated before processing for 2D staining as described later. This involved rehydrating samples in a methanol series (100%, 80%, 60%, 40%, 20%) and then washing in PBS twice.

Brain abbreviations are as follows: AUDd1, dorsal auditory area, layer 1; AUDp1, primary auditory area, layer 1; AUDpo1, posterior auditory area, layer 1; CA1, field CA1; COApl, cortical amygdalar area, posterior part, lateral zone; CP, caudoputamen; DG, dentate gyrus; ENT, entorhinal area; ENTl, entorhinal area, lateral part; ENTl1, entorhinal area, lateral part, layer 1; ENTl2, entorhinal area, lateral part, layer 2; ENTl5, entorhinal area, lateral part, layer 5; FRP, frontal pole; FRP2/3, frontal pole, layer 2/3; GU1, gustatory areas, layer 1; MOp, primary motor area; MOs1, secondary motor area, layer 1; ORB, orbital area; Pa4, paratrochlear nucleus; PAA, piriform-amygdalar area; PERIl6b, perirhinal area, layer 6 b; PIR, piriform area; PVH, paraventricular hypothalamic nucleus; RSP, retrosplenial area; sctd, dorsal spinocerebellar tract; SSp-m1, primary somatosensory area, mouth, layer 1; SSs1, supplemental somatosensory area, layer 1; SUB, subiculum; TEa1, temporal association areas, layer 1; TR, postpiriform transition area; VISal1, anterolateral visual area, layer 1; VISl1, lateral visual area, layer 1; VISp, primary visual area; VISp1, primary visual area, layer 1; VISpor1, postrhinal area, layer 1.

#### Preparation of brains for 2D staining

To cryoprotect brains before freezing, they were placed in a 15% sucrose/PBS solution overnight, followed by a 30% sucrose/PBS solution until they had dropped to the bottom of the tube. The brains were then coated in Tissue-Tek OCT compound, flash frozen and stored at −70°C until sectioning on a cryostat. Free floating sections (30 μm) containing the hippocampus and prefrontal cortex were collected (see Figure S4) and stored in cryoprotectant (PBS/ethylene glycol/glycerol, 2:1:1) at −20°C until staining.

#### Immunohistochemistry

To assess the potential anti-inflammatory actions of semaglutide and tirzepatide on gliosis in AD-relevant brain regions, microglia and astrocyte activation was examined via Iba1 and GFAP immunostaining, respectively. Brain sections were mounted onto slides with PBS and washed 3 × 10 min with 0.1 M Triton X-100/PBS (T-PBS) before blocking in 4% BSA/T-PBS for 1 h. Sections were then incubated with primary antibodies, diluted 1:500 in 1% BSA/T-PBS, overnight at 4°C (goat anti-Iba1 (Abcam, ab5076), rabbit anti-GFAP (Abcam, ab68428)). The next day, sections were washed 3 × 30 min with PBS and incubated with secondary antibodies, diluted 1:800 in 1% BSA/T-PBS, for 2 h at room temperature (AF donkey anti-goat 568 (Invitrogen, A-11057), AF donkey anti-rabbit 488 (Invitrogen, A-21206)). Following 3 × 30 min of washing with PBS, slides were coverslipped with Vectashield hardset mounting medium with DAPI (Vector Labs, H-1500-10) and stored at 4°C until imaging.

#### Thioflavin S staining

To complement 3D Aβ staining, plaques in the subiculum were also assessed with a thioflavin S stain. Brain sections, from the same brains previously used in 3D Aβ analysis, were mounted onto slides with PBS and washed 3 × 5 minutes with PBS. Sections were quickly rinsed in MilliQ water before a 10-min incubation in 1% thioflavin S solution (filtered immediately before use). To remove residual background stain, sections were washed 3 × 5 min with 70% ethanol, 3 × 3 minutes with 50% ethanol, 2 times with MilliQ water and once with 1*x* PBS. Finally, slides were coverslipped with Vectashield hardset mounting medium with DAPI (Vector Labs, H-1500-10) and stored at room temperature until imaging the following day.

#### Imaging and image analysis

All slides were imaged at 10x magnification on a Zeiss AxioImager M2 or AxioScan 7 and at least 6–10 whole brain sections per mouse, per region of interest were analyzed. ImageJ software was used for all image analysis.

Iba1 and GFAP images were initially analyzed by measuring the positive cell count normalized to the area of the region of interest for Iba1-positive microglia and GFAP-positive astrocytes within the subiculum (SUB), hippocampal formation minus subiculum (HPF), retrosplenial area (RSP), anterior cingulate area (ACA), and motor area (MO). Regions of interest were drawn manually using Allen’s Brain Atlas as a reference. Details on the sections used and regions of interest quantified relative to Allen’s Brain Atlas are in Figure S4.

Microglia morphology analysis was conducted on Iba1 positive cells (see Figure S5). First, the average soma size in terms of diameter was measured (μm) by tightly adjusting the image thresholding to only detect the cell somas. Next, measures including the longest shortest path (μm), number of branches, number of junctions and average branch length (μm) were calculated using the ImageJ analyze skeleton function on skeletonized Iba1 positive cells. Preprocessing steps were conducted before skeletonizing images using ImageJ functions such as subtract background, despeckle, adjusting min/max to ensure the entire cell including fainter processes could be thresholded, removing outliers, and selecting for particles of a given size to exclude double cells and cell fragments. See Figure S5 for example images at each stage of processing. Importantly, the same settings were applied across groups to all images in an experiment except in 5xFAD experiments where different thresholding was used for the WT group vs. all 5xFAD groups. This was because there was a stark difference in staining intensity and ensured the entire cell and its processes were thresholded.

Plaques stained positive with thioflavin S were measured as the number of plaques normalized to the area of the region of interest, the percent area of positive staining and the average plaque diameter (μm).

#### Gene expression analysis (qPCR)

The gene expression of different inflammatory and synaptic markers was examined in the hippocampus and prefrontal cortex of mice in cohort 1 of the 5xFAD study and in the LPS study.

For RNA extraction, hippocampus and prefrontal cortex tissues were homogenized in TRIzol reagent (QIAzol Lysis Reagent, Qiagen, 79306) with a 5 mm stainless-steel bead using a TissueLyser II (Qiagen). Total RNA was isolated using a QIAwave RNA Mini Kit (Qiagen, 74534), according to the manufacturer’s instructions.

For cDNA synthesis in 5xFAD experiments, total RNA was converted to cDNA by mixing with FS buffer (Thermo Fisher Scientific, 18080–044), dithiothreitol (Thermo Fisher Scientific, 18080–044), and random primers (Sigma-Aldrich, 11034731001) followed by incubation at 70°C for 3 min in a thermal cycler (Eppendorf Mastercycler Pro) for first-strand synthesis. dNTP (Thermo Fisher Scientific, R0192), RNaseOUT (Thermo Fisher Scientific, 10777019), and SuperScript III (Thermo Fisher Scientific, 18080–044) were subsequently added for cDNA synthesis using a thermal cycler (Eppendorf Mastercycler Pro) with the following steps: 5 min at 25°C, 60 min at 50°C, and 15 min at 70°C.

For cDNA synthesis in LPS experiments, total RNA was converted to cDNA by mixing with 5*x* iScript reaction mix and iScript reverse transcriptase (iScript cDNA synthesis kit, BioRad, 1708891) followed by 5 min at 25°C, 20 min at 46°C, and 1 min at 95°C in a thermal cycler.

cDNA was diluted 1:40 for all tissue and stored at −20°C. Gene expression was analyzed by quantitative polymerase chain reaction (qPCR). cDNA, primers (see Table S1), and PrecisionPLUS qPCR Master Mix with SYBRgreen (Primer Design) were mixed in a 384-well plate and incubated in a LightCycler (Roche, LightCycler 480 II) using the following steps: 2 min at 95°C, 45 cycles of 60 s at 60°C. The reference gene, *hprt1*, was used for all plates. Primers are listed in Table S1.

### Quantification and statistical analysis

#### Statistical analysis

Statistical analyses were performed using GraphPad Prism 10.6.1 (GraphPad) and figures were generated using GraphPad Prism or Biorender. Details of statistical analysis for each experiment are described in the figure legends. For comparison of multiple groups, one-way ANOVA with Tukey’s post-hoc test or two-way repeated measures ANOVA were used. For comparison of two groups, unpaired two-tailed t-tests with Welch’s correction were used. Comparison of the log_2_FC of Aβ coverage from preselected brain regions was done using one-way ANOVAs with Tukey’s post hoc multiple-comparison test relative to the WT vehicle, using a negative binomial generalized linear model to control for Gaussian distribution. All data are presented as mean ± standard error of the mean (SEM).

## Supplemental information

Document S1. Figures S1–S7 and Table S1

Document S2. Article plus supplemental information
