# Supplementary material for: Preventative semaglutide and tirzepatide treatment does not alter disease progression in the 5xFAD mouse model of Alzheimer's disease
Source: Cell Rep Med. 2026 Jul 7;7(7):102906. doi: 10.1016/j.xcrm.2026.102906 (PMC13400174; doi:10.1016/j.xcrm.2026.102906)
Supplement: Document S1. Figures S1–S7 and Table S1 [file mmc1.pdf]

Cell Reports Medicine, Volume 7

Supplemental information

Preventative semaglutide and tirzepatide treatment does not alter disease progression in the 5xFAD mouse model of Alzheimer's disease

Anika Vear, Sofie Amalie Olsen, Emilie Cathrine Holst Lange, Marie Amalie Müller, Charlotte Svendsen, and Christoffer Clemmensen

Supplemental information

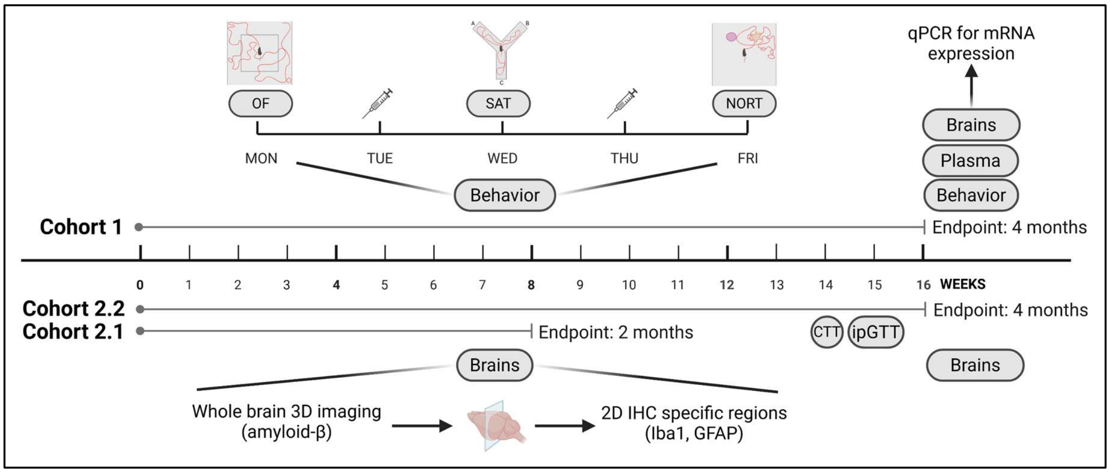

Figure S1. Overview of experimental design for 5xFAD study. Eight-week-old female WT or 5xFAD mice were treated on alternate days with either vehicle, semaglutide (10 nmol kg^{-1}) or tirzepatide (10 nmol kg^{-1}). The animals were divided into two experimental cohorts: (1) cohort 1 (n=12 per group) was treated for 4 months and underwent behavioral testing after 2- and 4-months of treatment, and (2) cohort 2 treated for either 2-months (cohort 2.1; n=8 per group) or 4-months (cohort 2.2; n=8 per group) underwent metabolic phenotyping including a compound tolerance test (CTT) and glucose tolerance test (GTT). Related to STAR Methods.

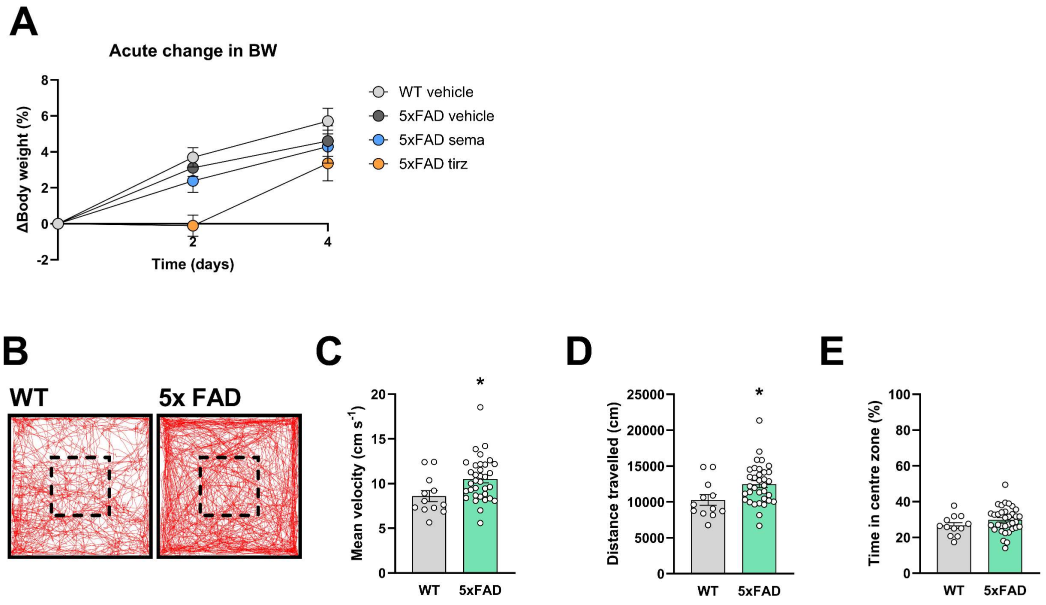

Figure S2. Acute body weight response to treatment and baseline open field test behavior. (A) Percent change in body weight from baseline after 2- and 4-days of treatment with either vehicle, semaglutide (10 nmol kg^{-1}) or tirzepatide (10 nmol kg^{-1}) in eight-week-old female WT or 5xFAD mice (n=28). Data is presented as mean ± SEM and was analyzed using a two-way repeated measures ANOVA. (B-E) Eight-week-old female WT or 5xFAD mice in cohort 1 underwent a 20-minute open field (OF) test at baseline. (B) Representative OF traces of a WT and 5xFAD mouse. (C) Mean velocity (cm s^{-1}), (D) distance travelled (cm) and (E) time spent in the center zone (%) were assessed. Data is presented as mean ± SEM. *$p<0.05$ vs WT via an unpaired t-test with Welch's correction (n=12-34). Related to Figure 1 and 2.

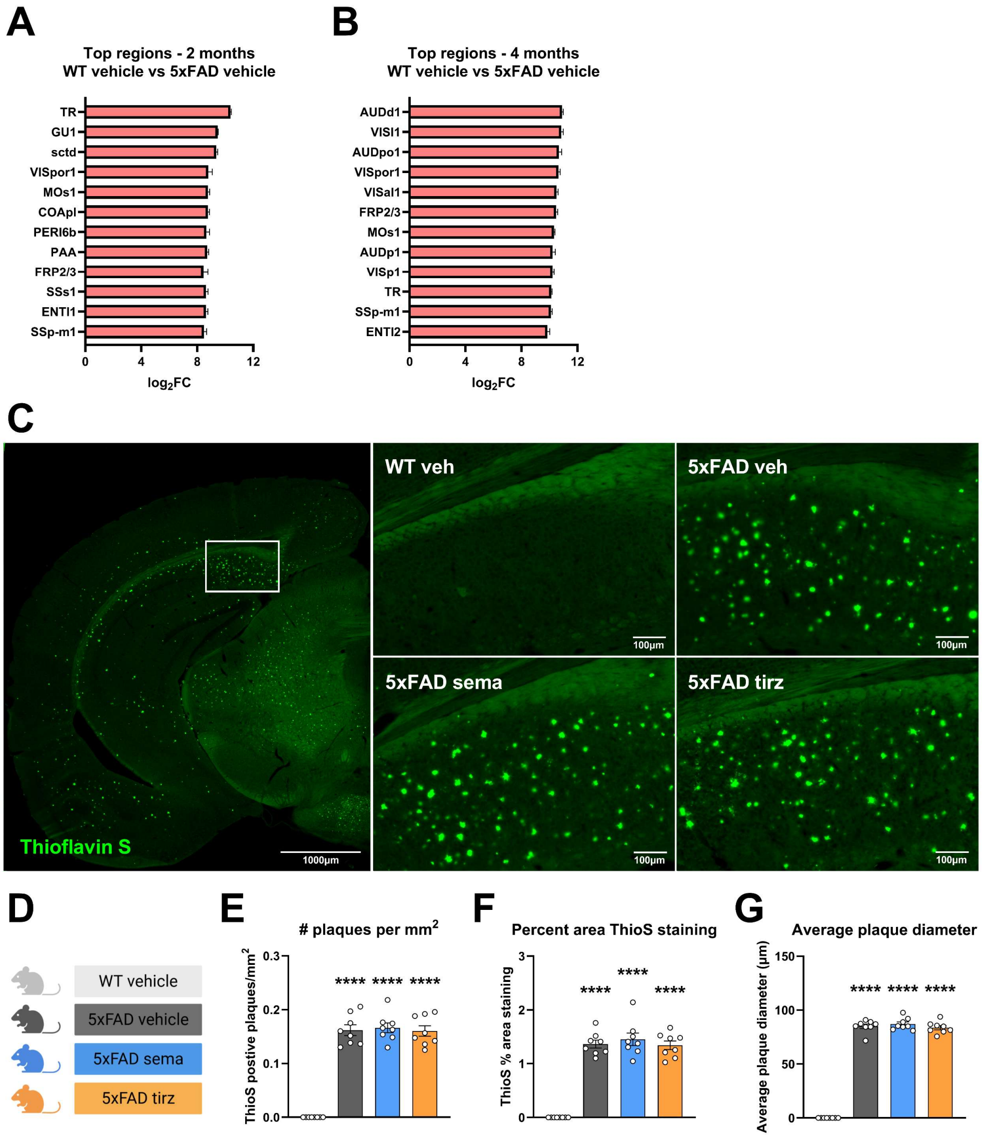

Figure S3. Extended Aβ analysis. (A,B) Top regulated brain regions (log_2FC) of the 5xFAD vehicle group vs the WT vehicle group in Aβ coverage (from 3D staining) after (A) 2- or (B) 4-months of treatment. Data is presented as mean ± SEM. Abbreviations for brain regions are defined in the methods. (C,D) Representative images of thioflavin S-positive plaques in the subiculum of WT or 5xFAD mice in cohort 2.1 treated on alternate days with either vehicle, semaglutide (10 nmol kg^{-1}) or tirzepatide (10 nmol kg^{-1}) for 2-months. The half brain section displayed is from a representative 5xFAD vehicle mouse and the zoom box highlights the region the representative images shown are from. (E-G) Quantification of the (E) number of thioflavin S positive plaques per mm^2, (F) percent area of positive staining and (G) average plaque diameter (μm) in the subiculum. Data is presented as mean ± SEM. ****$p<0.0001$ vs WT vehicle via one way ANOVA with Tukey's post-hoc test (n=8). Related to Figure 3.

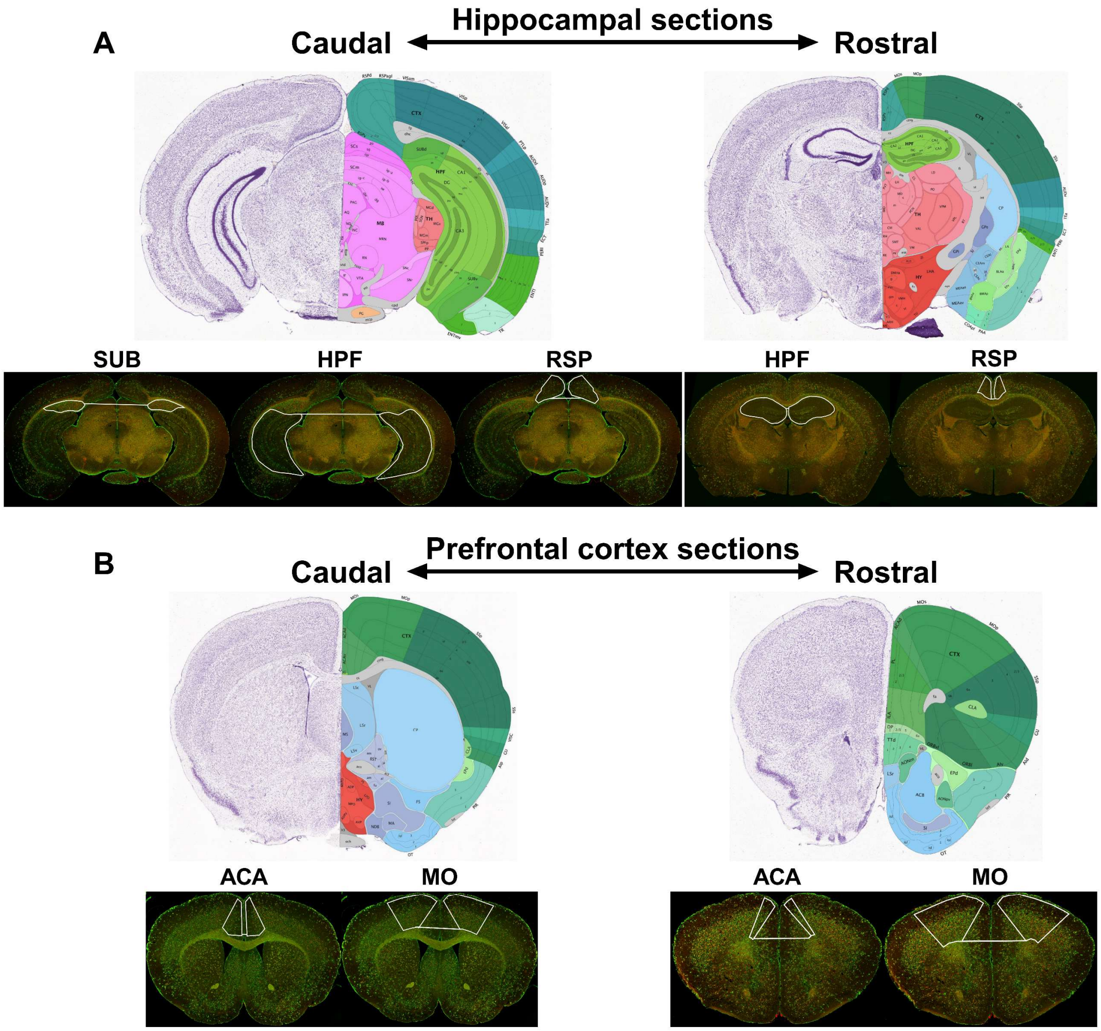

Figure S4. Regions of interest quantified in immunohistochemistry experiments. Upper panels, taken from Allen's Brain Atlas, show the outer range of sections (caudal to rostral) used for collection of the (A) hippocampus and (B) prefrontal cortex. Lower panels highlight representative regions of interest of the most caudal and rostral sections corresponding to the quantification of Iba1-positive and GFAP-positive cells. SUB, subiculum; HPF, hippocampal formation minus subiculum; RSP, retrosplenial area; ACA, anterior cingulate area; MO, motor area. Related to STAR Methods.

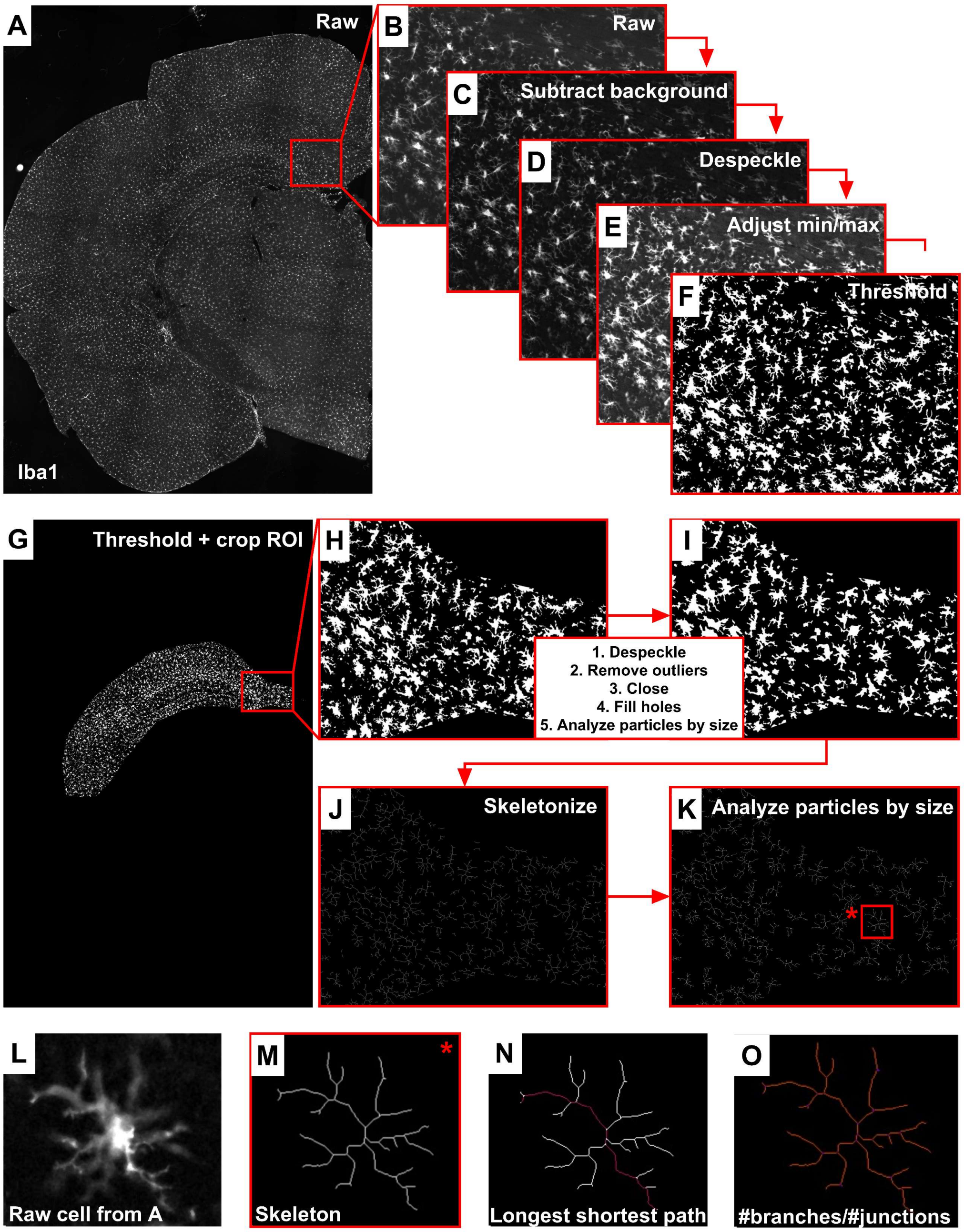

Figure S5. Image analysis pipeline of microglia morphology. Example images at each step of processing in the analysis of Iba1 positive microglia. (A) Raw unedited image of Iba1 stained microglia (10x magnification) in a half brain section from a representative LPS-treated mouse. Zoomed in image from the (B) raw image through each step of pre-processing including (C) subtract background, (D) despeckle, (E) adjust min/max and (F) threshold. (G) The thresholded binary image is cropped to remove staining outside the region of interest (ROI), here the hippocampal formation. (H) Zoomed in image before multiple processing steps including despeckle, remove outliers, close, fill holes and analyze particles by size, leaving the (I) final processed image. (J) The final processed image is skeletonized and then (K) particles are sorted by size to give skeletons of whole single cells. A single example cell is shown at the bottom in its (L) raw unedited form taken from A and (M) skeletonized form taken from K. Finally, each cell skeleton is analyzed including the (N) longest shortest path (in purple) and the (O) number of branches (in orange) and junctions (in purple). Related to STAR Methods.

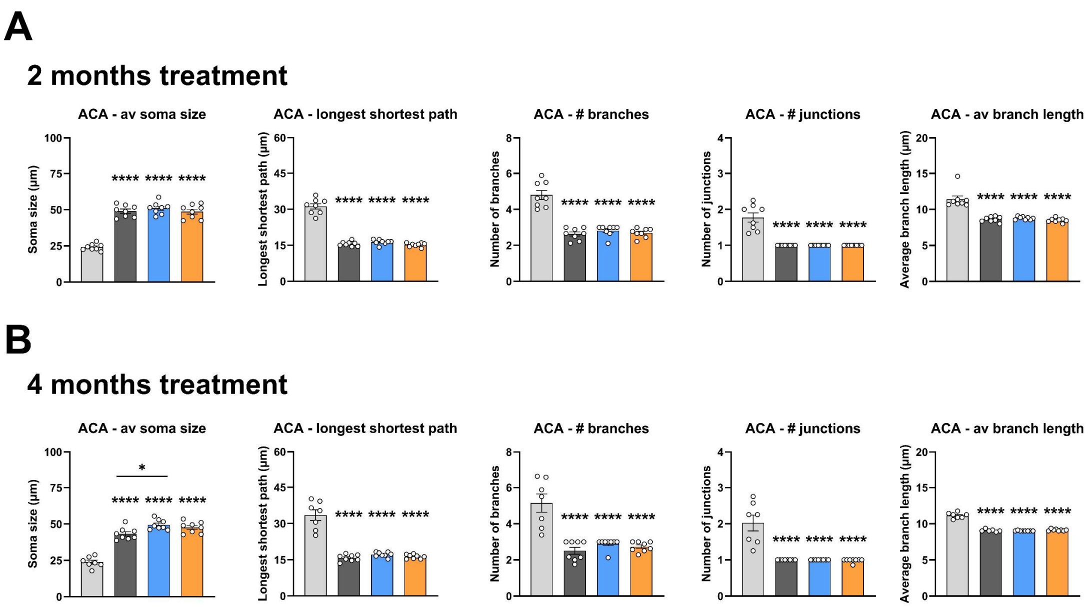

Figure S6. Morphological analysis of microglia in the anterior cingulate area of 5xFAD mice. Quantification of the morphology of Iba1 positive cells in the anterior cingulate area (ACA) of WT or 5xFAD mice in cohort 2.1 treated on alternate days with either vehicle, semaglutide (10 nmol kg^{-1}) or tirzepatide (10 nmol kg^{-1}) for (A) 2-months or (B) 4-months. Measurements include average soma size (µm), longest shortest path (µm), number of branches, number of junctions and average branch length (µm). Data is presented as mean ± SEM. ****p<0.0001 vs WT vehicle via one way ANOVA with Tukey's post-hoc test (n=7-8). Related to Figures 4 and 5.

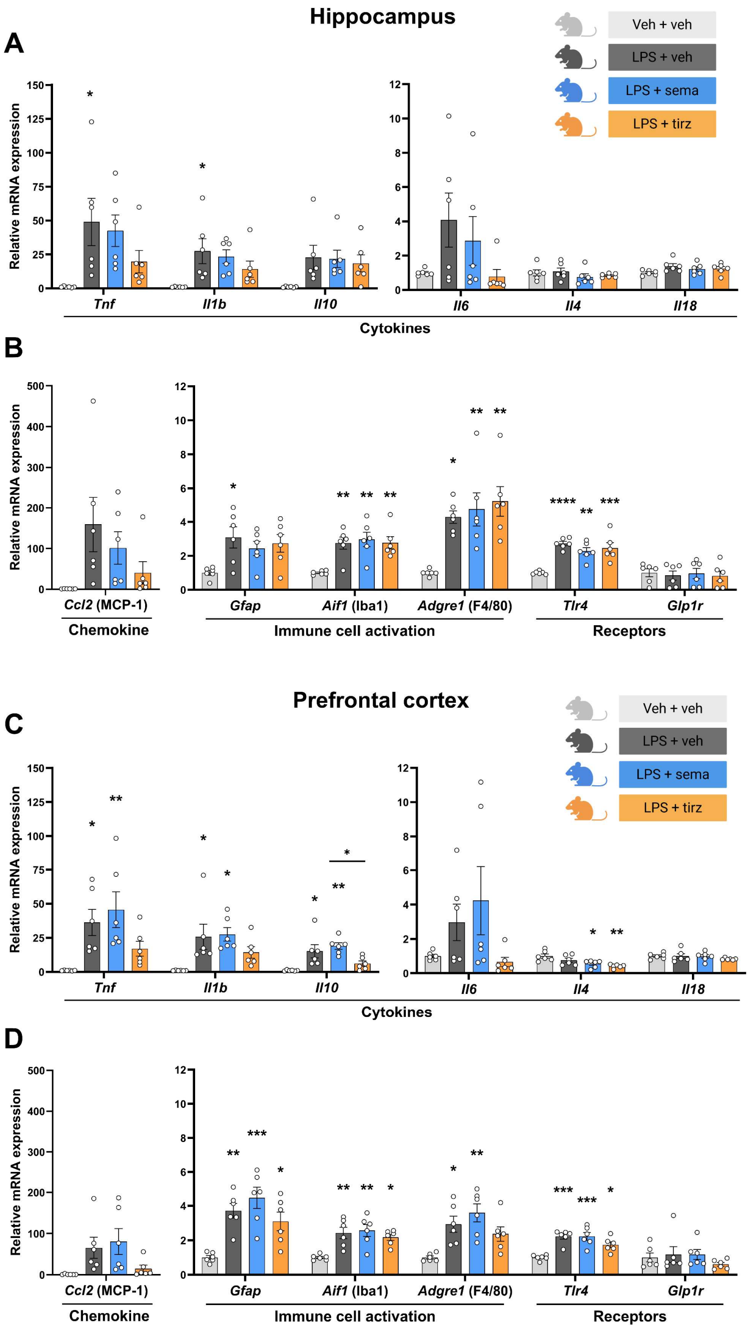

Figure S7. Regulatory role of semaglutide and tirzepatide on gene expression of inflammatory markers in LPS-treated mice. Brains were collected from male C57BL/6J mice that were treated daily for 3 days with either vehicle (veh), semaglutide (10 nmol kg^{-1}; sema) or tirzepatide (10 nmol kg^{-1}; tirz) followed by another 3 days of drug and either vehicle (veh) or lipopolysaccharide (250 µg kg^{-1}; LPS). Quantitative PCR analysis of transcript levels of a range of inflammatory markers such as (A,C) cytokines (*Tnf*, *Il1b*, *Il10*, *Il6*, *Il4*, *Il18*), (B,D) a chemokine (*Ccl2* (MCP-1)), immune cell activation (*Gfap*, *Aif1* (Iba1), *Adgre1* (F4/80)) and receptors of interest (*Tlr4*, *Glp1r*) in the (A,B) hippocampus and (C,D) prefrontal cortex are presented as mRNA expression relative to the mean of the veh + veh group. Data is presented as mean ± SEM. *$p<0.05$, **$p<0.01$, ***$p<0.001$, ****$p<0.0001$ vs veh + veh (unless otherwise specified) via one-way ANOVA with Tukey's post-hoc test (n=6). Related to Figure 7.

Table S1. Primers used for qPCR. Related to STAR Methods.

Symbol	Gene name	Forward sequence	Reverse sequence
Tnf	Tumor necrosis factor	GCCTCTTCTCATTCCTGC TTG	CTGATGAGAGGGAGGCC ATT
Il1b	Interleukin 1 beta	CACAGCAGCACATCAAC AAG	GTGCTCATGTCCTCATCCT G
Il6	Interleukin 6	TACCACTTCACAAGTCG GA	AATTGCCATTGCACAACT C
Il4	Interleukin 4	GATTCATCGATAAGCTG CACC	CATGATGCTCTTTAGGCTT TCC
Il10	Interleukin 10	CAGCCGGGAAGACAAT AACTG	CCGCAGCTCTAGGAGCAT GT
Il18	Interleukin 18	CCTTTGAGGAAATGGAT CCAC	GTCCTGGAACACGTTTCT G
Ccl5	C-C motif chemokine ligand 5	TCTTGCAGTCGTGTTTG TC	GTTGATGTATTCTTGAACC CAC
Ccl2 (MCP-1)	C-C motif chemokine ligand 2	GCATCCACGTGTTGGCT CA	CTCCAGCCTACTCATTGG GATCA
Map2	Microtubule associated protein 2	CGGAAAACCACAGCAG CAAG	GGGAGGATGGAGGAAGG TCT
Syp	Synaptophysin	CTGCGTTAAAGGGGGC ACTA	ACAGCCACGGTGACAAA GAA
Trp53 (p53)	Tumor protein p53	GTATTTCACCCTCAAGA TCC	TGGGCATCCTTTAACTCT A
Gfap	Glial fibrillary acidic protein	CGAAGAAAACCGCATC ACCAT	GTGGCCTTCTGACACGGA TT
Aif1 (Iba1)	Allograft inflammatory factor 1	CAAACTTGAAGCCTTCA AGG	CGCTTCAAGGACATAATA TCGA
Adgre1 (F4/80)	Adhesion G protein-coupled receptor E1	GTGCCATCATTGCGGGA TTC	AAGAGCATCACTGCCTCC AC
Cd68	CD68 molecule	ACAAAACCAAGGTCCA GGGA	ATTCTGCGCCATGAATGT CC
Tyrobp	Transmembrane immune signaling adaptor TYROBP	CACATTGCTGAGACTGA GTCGC	TTGAGGTCACTGTATACT TCTGGTCTCT
Clec7a	C-type lectin domain containing 7A	GCTCCCAGCTAGGTGCT CATC	TGTTTGGCTTTCAATGAA CTCAA
Tlr4	Toll-like receptor 4	GGCTAGGACTCTGATCA TGG	TTAGGAACTACCTCTATG CAGG
Glp1r	Glucagon-like peptide 1 receptor	CTAGGAACTCCAATATG AACTACTG	TGAAGATAAGAAAGTTGA CGCC
Hprt1	Hypoxanthine phosphoribosyltransferase 1	CTCATGGACTGATTATG GACAGGAC	GCAGGTCAGCAAAGAAC TTATAGCC
